# Health economic evaluations of digital health technologies—a rapid review of applied methods

**DOI:** 10.3389/fdgth.2026.1816757

**Published:** 2026-07-02

**Authors:** Alessandro Campione, Milan Zetzsche, Tanja Rombey, Hendrikje Rödiger, Cornelia Henschke

**Affiliations:** 1 Department of Health Care Management, Technische Universität Berlin, Berlin, Germany; 2 Institute of General Practice and Interprofessional Care, University Hospital Tübingen and Faculty of Medicine, Eberhard Karls Universität Tübingen, Tübingen, Germany

**Keywords:** digital health technologies, digital therapeutics, DiHA, economic evaluation, health economics

## Abstract

**Introduction:**

Digital health technologies (DHTs) offer significant potential to improve healthcare delivery, efficiency and outcomes. Despite increasing uptake, their full potential remains underused, partly because existing approaches and regulatory frameworks for generating economic evidence are not yet adequately adapted to the specific characteristics of DHTs.

**Methods:**

This rapid review examined methods applied in economic evaluations (EEs) of DHTs of studies published between 2020 and 2024. Following Cochrane guidelines, searches were performed in PubMed and Scopus, yielding 1254 unique records, of which 51 primary studies met inclusion criteria. Methodological characteristics, including general and EE-specific aspects, were stratified by EE type and underlying study design, distinguishing between EEs as a primary or secondary objective and model-based EEs. The included studies were assessed using RoB2, ROBINS-I, CHEQUE, and CHEERS-(AI).

**Results:**

Most EEs were based on randomized controlled trials, and employed conventional methods developed for non-DHT interventions. The included EEs showed considerable heterogeneity in perspectives, cost categories, and handling of uncertainty. Reporting and methodological quality were inconsistent, including incomplete descriptions of the DHTs. Key limitations included limited study registration and transparency, and bias from underlying studies, particularly when multiple data sources were used. Important DHT-specific aspects, such as technology life cycles and real-world transferability across user groups, were rarely addressed.

**Discussion:**

The findings highlight a fragmented landscape of methodological and reporting practices and underscore the need for adequately adapted, DHT-specific economic evaluation methods that better reflect the unique characteristics of DHTs and generate robust, decision-relevant evidence.

## Introduction

Digital health has emerged as a key future priority in healthcare (1), gaining momentum since the Covid-19 pandemic (2, 3). Scientific interest has expanded accordingly, as digital health technologies (DHTs) are increasingly evaluated in clinical trials (4). DHTs can enhance the quality and efficiency of care by supporting diagnosis, therapy, monitoring, and self-management (5–8). Advances in data-driven decision-making strengthen their potential to contribute to a more sustainable and equitable healthcare system (9, 10). Combined with emerging technologies, DHTs potentially offer economies of scale and high cost-effectiveness while maintaining individualized health services and enabling autonomous self-management (10–13). Current applications range from telemedicine (14, 15) and digital diagnostics to specific therapeutic interventions (16). Despite this potential, the generation of robust evidence for DHTs remains challenging. Existing approaches to evidence generation and evaluation, particularly in health economics, are not well adapted to the specific characteristics of DHTs, such as rapid innovation cycles, complex intervention components, and context-dependent effects (17–19). As a result, uncertainties persist regarding their value, comparability, and real-world impact, limiting their effective integration into healthcare systems and decision-making processes.

Regulatory approval in the United States and CE marking in the European Union define the conditions under which DHTs can enter the market, but they do not determine reimbursement or uptake in routine care. Coverage decisions are typically made by payers and health systems and increasingly require additional evidence, including clinical and economic evidence obtained from sources such as real-world data, clinical trials or meta-analyses. Different European health systems are adopting DHTs in clinical care, with varying reimbursement and assessment policies (20). Across countries, requirements for such evidence vary, with heterogeneous approaches to study design, comparators, and outcome measures (21–23). While several frameworks and taxonomies for assessing DHTs have been developed (24–26), they differ in scope, methodological rigor, and their integration into reimbursement processes.

Furthermore, patient adherence and adoption in Europe remain below expected levels (27, 28), partly due to a lack of regulatory requirements and national pathways for pricing and reimbursement (28). On the provider side, uncertainties regarding reimbursement models and shifting professional roles further hinder implementation (10, 29). These inconsistencies are particularly evident in the role and application of economic evaluations. While some countries, such as Belgium, require evidence of clinical or socio-economic added value linked to the care pathway and reflected in the comparator, they allow flexibility in study designs (23). In England, structured economic assessments are used to inform commissioning decisions, whereas Belgium and France do not specify economic evaluations (EEs) as requirements, only additional assessment domains (23). In Germany, no formal EE is required (23).

In addition, conventional methods originally developed for non-digital interventions, are often applied without adaptation, despite known challenges such as rapid technology cycles, difficulties in defining comparators, context-dependent effects, and limited generalizability. As a result, there is uncertainty about the methodological appropriateness and decision relevance of current economic evidence for DHTs (18, 19, 23, 30–33). Overall, the generation of robust evidence, such as EEs, remains challenging. Existing health technology assessment frameworks for DHTs are not unified (31, 32), guidance on evidence requirements is inconsistent (21, 32, 34) and the role of EEs in reimbursement decisions varies across countries (35). These factors create methodological and practical uncertainty for developers, evaluators and decision-makers and highlight the need for economic evaluation approaches that are adequately adapted to the unique characteristics of DHTs (4, 18, 19, 21, 36). To address this gap and to inform the development of evidence-based assessment approaches, this rapid review (RR) examines the methods used in EEs for DHTs, defined by the EU MDR, in English- and German-language research between 2020 and 2024. By systematically analyzing methodological approaches and their application, this review aims to identify common practices, limitations, and opportunities for improving the evaluation of DHTs.

## Methods

The RR was performed using an adaptation of the updated guidance on methods for Cochrane rapid reviews (37, 38). Reporting followed PRISMA guidelines (39), and the review was pre-registered on the OSF platform (40).

### Eligibility criteria

Patients and participants who received care that included the use of DHT were eligible if the DHT met the MDR definition (41). Any type of control group was eligible. To be included, studies had to analyze and report health economic outcomes. Model-based EEs and full EEs conducted in the context of clinical studies, comparative studies, controlled studies, randomized controlled trials, SLRs, or validation studies were eligible. Partial economic evaluations were not eligible, as they do not include a full comparison of both costs and outcomes (42). Studies included in SLRs had to meet these criteria and report a quality assessment. Studies limited to cost analyses, cost-minimization, or meta-analyses were excluded. Furthermore, we restricted inclusion to publications from 2020 to 2024 in English or German language.

### Information sources and search strings

A systematic literature search was conducted in PubMed and Scopus, with the most recent update in December 2024. Search strings were developed using a combination of keywords and MeSH terms. Full search strategies are provided in Supplementary Appendix 1. An additional unsystematic search based on the same search strings was conducted via the search engine Google.

### Selection process

Following the methods of the Cochrane rapid reviews methods group (37), one researcher (MZ) screened all titles and abstracts of the identified and deduplicated records. A second researcher (AC) independently screened a random 20% subsample for verification. Screening was performed using Rayyan (43). After title and abstract screening, MZ screened all full texts while AC screened any excluded full-text studies. Uncertainties or conflicts were resolved through discussion, until consensus was reached.

### Data extraction and data items

A standardized Excel spreadsheet was developed for data extraction. One reviewer (MZ) performed data extraction, which was verified by the second reviewer (AC). Extracted data items included the characteristics of the EE, technology details, costs, outcomes, methods, and results. For model-based EEs, model characteristics were additionally extracted.

### Synthesis method

Data were synthesized narratively and presented both in aggregated form and stratified into (i) studies in which the economic evaluation was the primary objective (EEs as a primary objective), and (ii) studies in which the economic evaluation was conducted as a secondary analysis (EEs as a secondary objective). Model-based EEs were analyzed as a separate category due to their distinct methodological approach. These strata were chosen to control heterogeneity and to provide a structured framework for synthesizing methodological approaches.

### Quality assessment

A combination of assessment instruments was used to evaluate the quality of included primary studies. Depending on the underlying study design, EEs were assessed using either the Risk of Bias 2 (RoB2) instrument for randomized controlled trials (RCTs) or the ROBINS-I instrument (44, 45) for non-randomized studies. All studies were also assessed using the CHEQUE (Domain M) (46). Reporting quality was assessed using the Consolidated Health Economic Evaluation Reporting Standards (CHEERS)-2022 (47) or the CHEERS-AI checklist (48) for interventions involving AI. This selection of tools ensured comprehensive assessment of relevant aspects of EEs while minimizing overlap. The CHEERS assessment was supported using AI, (ChatGPT, model GPT-o1 and o3-mini, OpenAI, San Francisco) using a comprehensive prompt (Supplementary Appendix 6) on each study. One researcher (MZ) verified accuracy of the outputs, using ChatGPT's clues to find the corresponding information in each study. Quality assessments were synthesized for each stratum and presented as an overview for each study.

## Results

### Study selection

**Figure 1:**
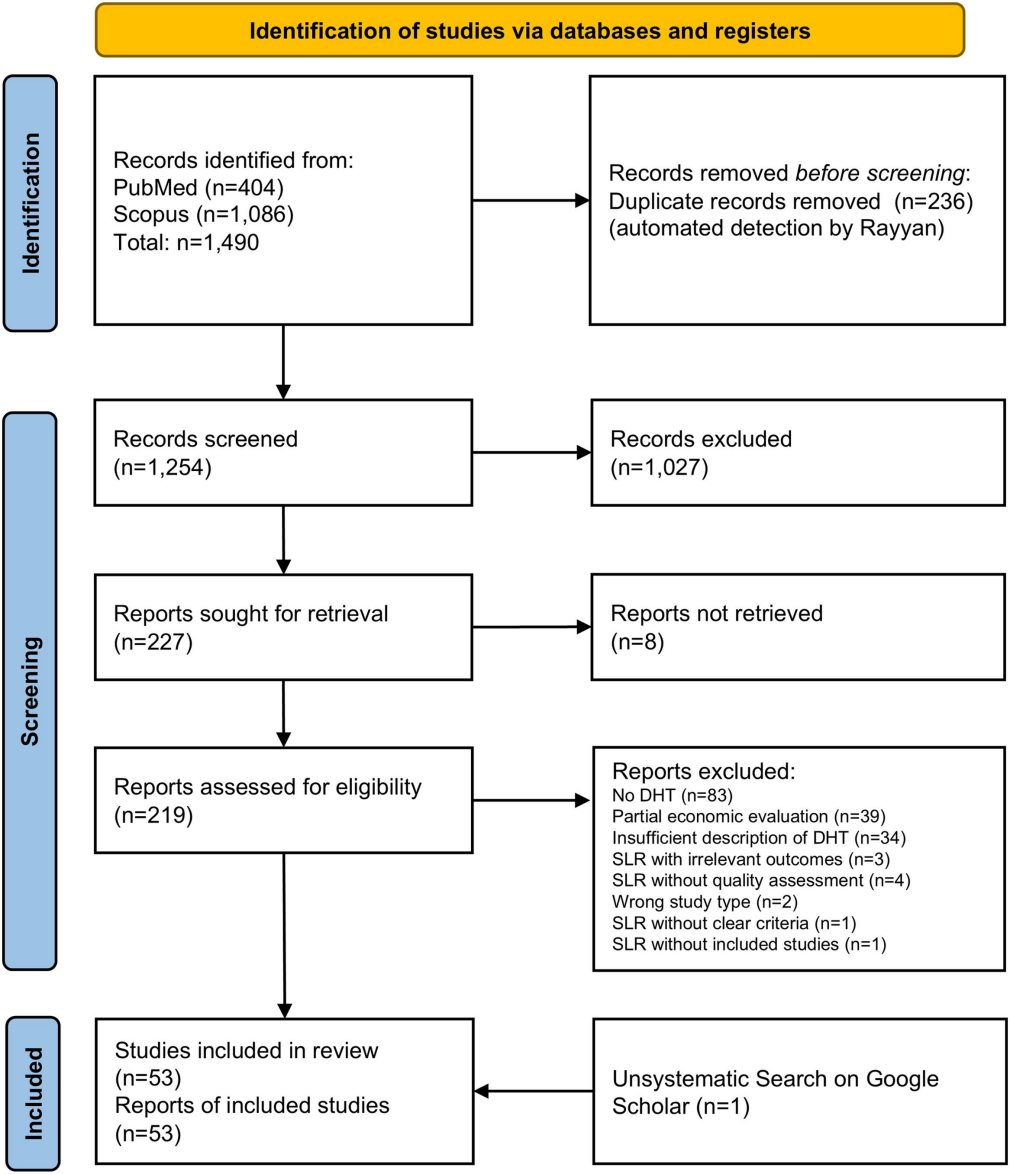
PRISMA 2020 flow-diagram.

A total of 1254 unique studies were included for title and abstract screening. A subsample of 20% (253 studies) was screened by a second reviewer (AC), resulting in 43 conflicts (17% of the subsample). All conflicts were explainable and could be resolved consensually. A total of 219 studies were subsequently eligible for full text screening. No full texts could be retrieved for 8 studies. A total of 169 full texts were excluded by the main reviewer (MZ). These were screened by AC, resulting in 18 conflicts. After deconfliction, 16 studies were excluded and 2 were included, leaving 52 studies. The Google search yielded one additional relevant study, which was included. Finally, 53 entries were included for synthesis, of which 51 were primary studies and two were systematic literature reviews (SLRs). The screening process is shown in Figure 1.

### Overview of characteristics of primary studies

Most of the 51 EEs originated from Europe (23 EEs), followed by North America (12 EEs). Publication years were almost equally distributed across the inclusion period. Most EEs analyzed samples between 100 and 499 participants (15 EEs), while nine were limited to sample sizes below 100. Hypothetical samples above 1,000 were analyzed in five EEs, and seven EEs did not report sample size. Most EEs applied a one-year time horizon (14 EEs), followed by hypothetical lifetime horizons (six EEs), long-term horizons of 5–10 years (four EEs), and multiple horizons (three EEs).

Use of guidelines or reporting recommendations was documented in 20 EEs, mostly referencing the Consolidated Health Economic Evaluation Reporting Standards (CHEERS). Nine studies applied multiple analytic perspectives. A societal perspective was used in 16 EEs, while 13 applied a payer perspective and 13 a health care provider perspective. In eight cases, the analytic perspective was not reported.

Cost-utility-analyses (CUAs) were the dominant type of analysis, performed in 31 studies. Cost-effectiveness analyses (CEAs) were performed in 10 EEs, while nine used a combination of CEA, CUA or cost-benefit-analysis (CBA). Among 29 EEs based on prior studies, EEs were the primary objective in 21 cases and a secondary objective in eight cases. Another 22 EEs were model-based. The underlying DHTs were classified according to their core technological features, with most assigned to mHealth and telemedicine (34 EEs), 12 to eHealth, and 5 to digital diagnostics.

An aggregate overview is provided in Table 1.

**Table 1:** Aggregated overview of characteristics of the *n* = 51 included evaluations.

Characteristic	Frequency [Percentage]
Region of origin	
* Europe*	23 [45.1%]
* North America*	12 [23.53%]
* Asia*	9 [17.65%]
* Oceania (Australia)*	6 [11.77%]
* Africa*	1 [1.96%]
Year of publication	
* 2020*	10 [19.60%]
* 2021*	12 [23.53%]
* 2022*	11 [21.57%]
* 2023*	9 [17.65%]
* 2024*	9 [17.65%]
Sample size	
* <100*	9 [17.65%]
* 100–499*	15 [29.40%]
* 500–1,000*	7 [13.73%]
* >1,000*	7 [13.73%]
* Hypothetical Cohort*	6 [11.76%]
* NA*	7 [13.73%]
Time horizon	
* Multiple*	*3 [5.88%]*
* <6 Months*	7 [13.73%]
* 6–<12 Months*	9 [17.65%]
* 1 Year*	14 [27.45%]
* >1–5 Years*	7 [13.73%]
* >5–10 Years*	4 [7.84%]
* Reported as lifetime*	6 [11.76%]
* NA*	1 [1.96%]
EE type	
* *CUA	31 [60.80%]
* *CEA	10 [19.61%]
* *CEA & CUA	7 [13.73%]
* *CBA	1 [1.96%]
* *CEA, CUA & CBA	2 [3.92%]
BIA*	*2*
Technology category	
* *mHealth	17 [33.33%]
* *Telemedicine	17 [33.33%]
* *eHealth	12 [23.53%]
* *Digital Diagnostics	5 [9.80%]
Intervention aim	
* *Prevention	3 [5.88%]
* *Treatment	22 [43.14%]
* *Rehabilitation	6 [11.75%]
* *Monitoring/Surveillance	12 [23.53%]
* *(Self)management	8 [15.68%]
Targeted disease/condition	
* *Cardiovascular diseases	13 [25.50%]
* *Psychiatric conditions	7 [13.72%]
* *Oncologic diseases	5 [9.80%]
* *Other chronic diseases	24 [47.06%]
* *Post-operative orthopedic conditions	1 [1.96%]
* *Problematic alcohol use	1 [1.96%]

NA, not available; CUA, cost-utility analysis; CEA, cost-effectiveness analysis; CUA, cost-utility analysis; CBA, cost-benefit analysis; *BIA, budget-impact analysis counted separately.

Table 2 summarizes EE-specific characteristics across the three EE-groups. Analytic perspectives were similarly distributed between groups, however, EEs as a secondary objective often did not report sufficient information. Loss of productivity costs were underrepresented in EEs as a secondary objective, while nearly all model-based EEs measured direct medical costs (DMC) and intervention costs. The bottom-up approach was common, although the specific calculation method was often unreported in model-based EEs and those conducted as a secondary objective. Probabilistic sensitivity analysis (PSA) was most frequently applied in model-based EEs to account for uncertainty, while EEs as a primary objective used bootstrapping more often. Model-based EEs also showed greater variation in the presentation of results, including the use of graphical outputs such as tornado diagrams. EEs as a secondary objective had the highest share of approaches addressing heterogeneity or distributional effects and most frequently applied scenario analysis. However, no alternative analyses were performed in this group. Details of EE-specific characteristics are found in Supplementary Appendices 2.1, 3.1, 4.1 and are discussed per group in the following sections.

**Table 2:** Overview of economic-evaluation-specific characteristics stratified by economic evaluation type.

EE-specific characteristics	Characteristic	EEs as a primary objective (*n* = 21)	EEs as a secondary objective (*n* = 8)	Model based EEs (*n* = 22)
Perspectives*	Societal	9 (49–57)	1 (58)	8 (59–66)
	Health care provider	5 (53, 67–70)	—	3 (66, 71, 72)
	Health care system	3 (52, 73, 74)	—	3 (62, 75, 76)
	Payer	2 (77, 78)	2 (79, 80)	8 (81–88)
	Patients	2 (68, 73)	1 (80)	—
	Decision maker in health care system	1 (89)	—	1 (90)
	Employer	2 (57, 91)	—	—
	(Personal) social services	—	1 (80)	1 (82)
	NR/unclear	2 (92, 93)	5 (94–98)	1 (99)
Outcome*	QALY	18 (49–57, 67–70, 73, 74, 77, 89, 91)	4 (58, 80, 94, 97)	20 (59–66, 71, 72, 76, 82–88, 90, 99)
	Natural unit	6 (50, 74, 78, 91–93)	3 (80, 98)	5 (63, 64, 75, 81, 99)
	Other	2 (57, 69)	4 (79, 95–97)	—
Costs*	DMC	19 (49–57, 67–70, 73, 74, 78, 89, 92, 93)	8 (58, 79, 80, 94–98)	22 (59–66, 71, 72, 75, 76, 81–88, 90, 99)
	DnMC	12 (49, 51, 52, 54–57, 68, 73, 74, 91, 92)	2 (58, 80)	7 (59, 62–64, 66, 75, 90)
	Loss of productivity	8 (50–53, 55–57, 73)	1 (79)	5 (59, 63–65, 90)
	Intervention costs	15 (49–51, 53–56, 68, 70, 74, 77, 78, 89, 91, 93)	5 (58, 80, 95, 96, 98)	21 (59–66, 71, 75, 76, 81–88, 90, 99)
Calculation method*	Bottom-up approach	19 (49–57, 68–70, 73, 74, 77, 78, 89, 91, 92)	3 (80, 95, 96)	5 (62, 65, 66, 82, 83)
	Top-down approach	2 (67, 93) and 2 (*intervention costs only*) (51, 55)	2 (79, 98)	1 (76)
	Mixed	—	1 (58)	2 (60, 85)
	Unclear/NR/NA	—	2 (94, 97)	14 (59, 61, 63, 64, 71, 72, 75, 81, 84, 86–88, 90, 99)
Accounting for uncertainty (Type)*	Parameter	5 (53, 67, 74, 89, 91)	3 (58, 79, 80)	10 (61, 63–65, 71, 76, 83–85, 90)
	Model uncertainty	—	—	1 (72)
	NR	15 (49–52, 54–57, 68–70, 73, 77, 78, 93)	5 (94–98)	11 (59, 60, 62, 66, 75, 81, 82, 86–88)
	None	1 (92)	—	1 (99)
Method of accounting*	SAS/DSA	8 (50, 51, 53, 55, 57, 68, 74, 89)	1 (80)	14 (59–64, 66, 75, 76, 84, 86–88, 90)
	Scenario analysis	—	3 (58, 95, 96)	2 (62, 84)
	Bootstrapping	14 (50, 51, 53–57, 67–70, 74, 91, 93)	5 (79, 80, 94, 97, 98)	
	PSA	5 (49, 52, 73, 77, 89)	—	17 (59–65, 71, 72, 76, 81–86, 90)
	Other methods	2 (69, 78)	—	—
	None	1 (92)		1 (99)
Graphical presentation of results*	CEP	16 (49–56, 67–70, 74, 89, 91, 93)	2 (79, 80)	14 (60–66, 71, 72, 82–84, 86, 90)
	CEAC	12 (49, 50, 52, 53, 56, 57, 67, 69, 70, 74, 77, 89)	3 (80, 97, 98)	12 (59, 60, 62–65, 71, 76, 81, 84, 86, 90)
	Other	—	1 (94)	11 (61–64, 71, 76, 83–86, 90)
	None	4 (73, 78, 89, 92)	3 (58, 95, 96)	4 (75, 87, 88, 99)
Heterogeneity/distributional effects	Heterogeneity or distributional effects	2 (53, 74)	5 (58, 79, 80, 97, 98)	3 (60, 61, 84)
	None	19 (49–52, 54–57, 67–70, 73, 77, 78, 89, 91–93)	3 (94–96)	19 (59, 62–66, 71, 72, 75, 76, 81–83, 85–88, 90, 99)
Alternative analyses	INMB/NMB/BIA	5 (52, 56, 69, 78, 91)	—	7 (61, 62, 72, 81, 83, 85, 86)
	None	16 (49–51, 53–55, 57, 67, 68, 70, 73, 74, 77, 89, 92, 93)	8 (58, 79, 80, 94–98)	14 (59, 60, 63–66, 71, 75, 82, 84, 87, 88, 90, 99)

*Multiple entries per study possible.

INMB, incremental net monetary benefit; NMB, net monetary benefit; BIA, budget impact analysis; CEP, cost-effectiveness plane; CEAC, cost-effectiveness acceptability curve; SAS, sensitivity analysis; DSA, deterministic sensitivity analysis; NR, not reported; NA, not applicable; DMC, direct medical costs; DnMC, direct non-medical costs; QALY, quality-adjusted life years. EEs as a primary objective: studies in which the economic evaluation was the primary objective. EEs as a secondary objective: studies in which the economic evaluation was conducted as a secondary analysis.

### Economic evaluations as a primary objective

In this stratum, 18 out of 21 EEs were based on previously conducted RCTs. One study was based on a controlled clinical study, without randomization or blinding (73), another on a controlled non-inferiority design without randomized allocation (92), and a third on a pilot RCT with limited reporting (70). A total of 13 EEs were conducted as CUA, three deployed CEA and another three combined of CEA and CUA. Two EEs included CBA in addition to CEA and CUA and (57, 91). Intervention aims covered prevention (74, 78), treatment (53, 67, 69, 77, 91, 93), rehabilitation (54, 68, 89), monitoring/surveillance (49, 52, 56, 73) and (self)management (50, 51, 55, 57, 70, 92).

All interventions were delivered in a home setting. Furthermore, 11 interventions comprised different components, rather than stand-alone solutions, for example, a fall prevention intervention supplemented with additional aids (74) or two interventions using multiple monitoring devices (89, 93). Five studies assessed true stand-alone interventions (50, 53, 54, 56, 91). In four of these studies, a stand-alone intervention was compared to standard care (51, 55, 78, 93). Overall, standard care was the comparator in 18 studies, while three studies did not define the comparator as standard care (50, 67, 91). Of these, one compared moderated online discussion boards against behavioral therapy (67) while the second compared the intervention against a non-interactive information brochure (50), and the third used one-to-one and group courses (79). In total, two EEs compared an intervention using two control groups (57, 91). The interventions covered diverse chronic conditions, including lower back pain, diabetes or arthritis (49, 54, 56, 69, 70, 73, 77, 91, 92), cardiovascular diseases (52, 67, 73, 89, 93), oncologic conditions (51, 53, 55), psychological conditions (50, 78), orthopedic conditions (68), and problematic alcohol use (57). A societal perspective was most common (nine EEs), followed by health provider (five EEs) and health-system perspectives (three EEs). Two EEs each applied a payer (77, 78), employer (57, 91), or patient perspective (68, 73), while one used a health care decision maker perspective (89). In two cases, the perspective was not reported, although the included costs indicated either a societal (92), a health care system, or a payer perspective (93).

In total, 19 EEs used a bottom-up approach to measure resource use at the patient-level. Intervention costs in these studies can be categorized into hardware costs (50, 51, 54, 55, 70, 74, 77, 78, 93), licensing costs (49, 56, 68, 78, 89, 91) and hosting/maintenance/support (49–51, 53, 55, 91, 93). Human resource costs showed high heterogeneity, ranging from installation and patient visit expenses (74) to costs for recruiting and coaching of patients (78). Cost data were primarily sourced from study documentation and protocols (50, 51, 53–55, 57, 68–70, 74, 92), followed by hospital/department data (52, 53, 56, 70, 73, 92, 93), surveys (49, 52, 56, 68, 73, 91) and electronic patient documentation (56, 67, 78, 89).

Quality adjusted life years (QALYs) were the most common outcome (18 EEs), in most cases generated through EQ-5D-5L- or -3L instruments. Some exceptions used alternative questionnaires (70, 74, 91). Most CUAs used the area-under-the-curve method, with exceptions where instruments lacked preference weights [e.g., St. George's respiratory questionnaire (77)]. Six CEAs reported natural outcome units such as depression free days, number of falls or number and duration of hospitalizations. Two EEs used horizons longer than one year and applied accounting (73, 74). All but one study (92) reported methods to address uncertainty. Bootstrapping was most common, while some studies deployed probabilistic sensitivity analysis (PSA) with Monte-Carlo simulation (MCS) (73, 77, 89) or bootstrapping (49, 52). EE-specific and general characteristics are summarized in Supplementary Appendices 2.1 and 2.2.

### Economic evaluations as a secondary objective

In total, eight EEs were conducted as a secondary objective. Among this stratum, one publication constituted a special case of a comprehensive health technology assessment (HTA) that included an RCT (80). Three EEs were conducted alongside RCTs (58, 79, 97), two alongside implementation studies (95, 96), one along a randomized experimental study (94) and another alongside a retrospective, population-based study (98). CEAs were most common, accounting for five EEs (79, 95–98). Furthermore, one EE used a combination of CEA and CUA (80), while one conducted CUA (94) and one a CBA (58).

In five out of the eight EEs, the intervention goal was therapy, including three targeting chronic diseases (80, 95, 96) and two addressing lower back pain (79, 94). Further intervention goals included surveillance of patients with chronic diseases (58), management and monitoring of hypertension (97), and skin cancer detection (98). Stand-alone interventions were identified in three EEs, while the other five assessed DHTs with additional devices. All eight EEs investigated interventions deployed in a home setting and used standard care as the comparator.

Two EEs used a societal or payer perspective, and one applied the perspective payers, patients and “personal social services” (80). In five EEs the perspective was not reported. Overall, measured intervention costs were heterogeneous, yet three EEs did not evaluate these costs and two lacked reporting. Diverse direct non-medical costs (DnMC), such as travel expenses, patient membership costs, and child care costs were gathered in one EE (80), while a recent EE was the only study among all included accounting for taxation (58). Three EEs used a bottom-up-approach for measuring resource use and costs (80, 95, 96), while two used top-down approaches (79, 98). Sources of resource data included study documentation (58, 80, 95) sickness fund data (79, 98), patient charts (95), and patient surveys (58). DMC were mentioned in one EE, yet no details were reported (94). In two EEs, the data sources could not be identified (96, 97).

Health outcomes in CUAs were primarily measured in QALYs, using questionnaires such as the EQ-5D-5L (80) or the SF-12 (94). The EQ-5D-5L was also applied within a CBA (58). In two CUAs, health-related quality of life (HRQoL) was measured using the SF-12 (95, 96) but the results were not converted into utility weights. Additional CEA outcomes included minutes of physical activity measured by accelerometer (80), reduced pain on a numeric scale (97), and number of identified skin lesions (98). None of the studies in this stratum applied discounting, as time horizons did not exceed a year. Uncertainty and heterogeneity were addressed in various ways, with several EEs examining parameter uncertainty (58, 79, 80). Three EEs performed bootstrapping to estimate uncertainty via cost-effectiveness planes (CEP) or cost-effectiveness acceptability curve (CEAC) (79, 80, 97). One EE used bootstrapping to construct confidence intervals without further analysis (94). Scenario analyses were performed in three EEs (58, 95, 96), though the latter two limited variation to intervention costs. EE-specific and general characteristics are summarized in Supplementary Appendices 3.1 and 3.2.

### Model-based economic evaluations

A total of 22 EEs were model-based, referring to analyses based on mathematical modelling. Of these, 14 EEs drew on multiple sources of information, while eight relied on a single source (eight EEs). Most EEs deployed CUA (17 EE). Three reported a combination of CEA and CUA (63, 64, 99), and two used CEA exclusively (75, 81).

Intervention goals of model-based EEs included therapy (61, 65, 66, 71, 75, 81–84, 87, 88), rehabilitation (72, 85, 90), screening (59, 63, 64, 76, 99), self-management (86), prevention (62), and monitoring (60). Stand-alone interventions were assessed in seven EEs, while another six required additional devices. In seven EEs, the main application was used along standard therapy. Two interventions consisted of the central DHT in combination with a web- or eHealth tool (84, 99). One EE of an AI screening tool compared autonomous and assistive AI modes against face-to-face screening as well as telemedicine screening (76). The diseases under intervention included cardiovascular diseases (60, 71, 72, 75, 85, 90, 99), diabetes and related complications (61–64), and other chronic diseases (65, 66, 76, 84, 86). Psychiatric conditions were analyzed in five EEs (81–83, 87, 88), and one EE examined oncologic diseases (59).

The payer and societal perspectives were most frequently applied (eight EEs each), followed by the health care provider perspective (three EEs) and the health system perspective (three EEs). One EE combined multiple perspectives without providing separate analyses (82), one applied the perspective of health system decision makers without defining the term (90), and one EE did not report the perspective (99). Seven EEs used DnMC, primarily transportation costs (62–64, 75). One EE included time spent by patients and nursing (66), and another included living and housing costs (59). Valuation for DnMC was reported in one EE without further explanation (90). Intervention costs were defined as usage fees for the DHT in ten EEs, while four EEs did not provide details on these costs (59, 60, 62, 63). Two EEs limited intervention costs to implementation costs (66, 71). Three studies included multiple intervention costs (64, 85, 86), while one EE did not include intervention-specific costs at all (72). Loss of productivity was measured as income loss in two EEs (63, 64).

Resource use was retrieved from multiple secondary sources in 15 EEs. Some studies surveyed hospital cost data (59, 72, 81, 82), while three relied on study documentation (64, 85, 86). One EE used patient files (90), and another used patient surveys (82). Market values for intervention costs were calculated in six EEs, and some studies consulted public price catalogues (65) or official catalogues (64). In one EE, the authors conducted own pooled-analyses (62).

As most model-based EEs were CUAs, QALYs were calculated in 20 EEs. The EQ-5D-5L was the most frequently used instrument, serving either for health-state measurement (five studies) or for valuation procedures to generate utility values (six studies). Cost and outcomes were discounted in 13 EEs, as model-based analyses used longer time horizons. Discount rates ranged from 0.4% (83) to 5% (85, 99).

Markov models were the most frequently used modelling approach, applied in 14 EEs. Cycle lengths were one month (six EEs), three months (one EE), or 12 months (six EEs), with models simulating between three and seven states. Special cases included Markov models with half-life-correction (83) and a bivariate Markov model with MCS (82). Deterministic point estimates of costs, outcomes, and transition probabilities were used in 12 EEs. Two Markov models deployed MCS (65, 84), while decision trees were modelled in five EEs. Only ten EEs explicitly mentioned and addressed uncertainty of parameters and structure. One EE mentioned model uncertainty and addressed it using PSA (72), though only parameter uncertainty was considered (100). Ten studies mentioned parameter uncertainty, while eleven reported no information, despite ambiguous methodological descriptions.

EE-specific and general characteristics are summarized in Supplementary Appendices 4.1 and 4.2.

### Quality assessment

#### Reporting quality [CHEERS-(AI)]

CHEERS-average reporting quality across all studies was 71.81%, while the five studies involving AI had an average of 62.72%. Reporting quality assessments showed certain items being consistently underreported, such as the development of a health economic analysis plan. However, this item was just recently included in the 2022 version of CHEERS (Item 4). Settings and locations were also frequently unreported across all types of EEs (Item 6). In some cases, the intervention strategy and the rationale for its selection were not reported (Item 7). Methods for assessing subgroup impacts or distributional effects across population groups were reported only in a minority of EEs (Items 18, 19). Similarly, few EEs discussed differences between user groups or stakeholder engagement in their studies (Item 25). While modeling- and statistics-related items were often not applicable to EEs conducted as a primary objective, important details remained underreported in some cases (Items 16, 17). Dates of estimated resources, currency, and years of conversion were less frequently specified in EEs as a primary or secondary objective (Item 15).

#### Risk of bias of randomized studies (RoB2)

Across all EE-types, the most common sources of RoB (risk of bias) were deviations from intended interventions and missing outcome data. Some concern was apparent in the selection of reported results, as only few studies reported usage of pre-registered analysis plans. In contrast, RoB related to outcome measurement or randomization was rarely a concern. Overall, 18 EEs were assessed as having high RoB, while 17 EEs were judged to have some concerns according to RoB2.

#### Risk of bias of non-randomized studies (ROBINS-I)

All but one non-randomized study were assessed as having critical RoB under ROBINS-I, mostly due to lack of control for confounding factors, classification of interventions, or missing data. Most non-randomized studies did not sufficiently attempt to adjust for potential and significant confounding and were hence assessed at critical risk from ROBINS-I pre-assessment.

Seven model-based EEs could not undergo RoB2 or ROBINS-I assessment as the primary underlying study was not reported or discernible (59, 60, 62–64, 76, 83). For example, one study performed a pooled-meta analysis of multiple studies to estimate effects for modelling (62).

#### Methodological study quality (CHEQUE)

Critical CHEQUE (Domain M) assessment items across all EE types included consequences of choice of intervention and comparison (M17), adjustment of health preferences (M18), or a lack of probabilistic sensitivity analyses to account for uncertainty (M22). Only a minority of EEs explored alternative modeling assumptions to explore structural uncertainty or discussed equity-relevant or distributional considerations (M23). Model-based studies specifically lacked model validation and an appropriate approach to select data sources for model-parameters (M13,14).

No scoring tool is yet available to summarize findings across the different instruments, showing the need for a unified assessment tool of DHTs. Some EEs showed that even strong methodological quality and good reporting may be undermined by the quality of the underlying study, introducing bias into the EEs. Table 3 shows a summary of the assessments, while details on the assessment items are available in Supplementary Appendix 5.

**Table 3:** Summary of quality assessments.

Stratum	EE first author	CHEERS-(AI)	RoB Overall	ROBINS-I	CHEQE (Domain M) Out of 100 points (NAs counted as 0 points*)
EEs as primary objective (21)	Mujcic 2022 (50)	85.71%	High Risk		60
	Schuit 2022 (55)	78.57%	Some concern		52.5
	Pelle 2022 (69)	78.57%^†^	High Risk		55
	Lopez-Villegas 2020 (52)	82.14%^†^	Some concern		54
	Ambrens 2022 (74)	89.29%^†^	Some concern		52.5
	Nelson 2021 (68)	82.14%	Some concern		52
	van der Hout 2021 (51)	89.29%	Some concern		51.5
	Bernard 2022 (49)	64.29%^†^	Low risk		51
	de Jong 2020 (56)	75.00%^†^	Some concern		49
	Mudiyanselage 2023 (70)	75.00%	Some concern		49
	Mourad 2022 (67)	60.71%	High risk		49
	Buntrock 2021 (57)	78.57%^†^	Some concern		48
	Dawkins 2024 (53)	78.57%^†^	High risk		48
	Boggs 2022 (78)	71.43%	Some concern		46
	Dagenais 2021 (91)	75.00%	Some concern		47.5
	Liu/Tang 2023 (89)	64.29%	Some concern		47
	Ney 2021 (77)	53.57%	Some concern		44.5
	Fatoye 2020 (54)	78.57%	High risk		43.5
	Lam 2024 (93)	53.57%	Some concern		43.5
	Bautista-Mesa 2020 (73)	75.00%		Critical risk of bias	53.5
	Lemelin 2020 (92)	50.00%		Critical risk of bias	21.5
EEs as a secondary objective (8)	Taylor 2020 (80)	89.29%^†^	Some concern		58
	Priebe 2024 (79)	71.43%	High risk		55.5
	McManus 2021 (97)	64.29%	High risk		46
	Sten-Gahmberg 2024 (58)	82.14%^†^	High risk		42.5
	Park 2023 (94)	44.74%	High risk		31
	Smak-Gregoor 2023 (98)	57.89%		Moderate risk of bias	44
	Colomina 2021 (96)	64.29%		Critical risk of bias	41
	De Batlle 2021 (95)	67.86%		Critical risk of bias	40.5
Model-based EEs (22)	Luo 2022 (71)	71.43%^†^	High risk		91
	Park 2024^a^ (62)	78.57%^†^			90
	Lewkowicz 2023 (65)	75.00%	High risk		88.5
	Senanayake 2023 (85)	75.00%^†^	Some concern		86
	Freitag 2024^b^ (83)	78.57%^†^			85.5
	Nomura 2022 (84)	75.00%^†^	Some concern		84
	Liu/Zhan 2023 (90)	64.29%	High risk		84
	Davison 2024 (61)	78.57%^†^	High risk		83.5
	Caillon 2022^c^ (60)	78.57%^†^			77.5
	Bhardwaj 2021 (72)	78.58%		Critical risk of bias^+^	69.5
	Miranda 2022 (86)	71.43%	High risk		62.5
	Morrison 2022 ^d^ (76)	71.05%			69
	Velez and Malone 2021 (87)	67.86%	High risk		62.5
	Velez 2021 (88)	64.29%	High risk		62.5
	Patel 2020 (75)	67.86%^†^	High risk		61
	Lin 2024^e^ (63)	73.68%			60
	Lin 2023^e^ (64)	76.32%			60
	Piera-Jiménez 2020 (66)	75.00%^†^		Critical risk of bias^+^	55.5
	Greenwood 2024 (82)	82.14%^†^	Some concern		55
	Wang 2021 (81)	64.29%	High risk		49
	Zhang 2023^f^ (59)	53.63%			47
	Orchard 2020 (99)	42.86%		Critical risk of bias	47.5

NA, not applicable. If the study used multiple sources (especially for model-based EEs), the primary RCT was assessed if disclosed as such. EEs as a primary objective: studies in which the economic evaluation was the primary objective. EEs as a secondary objective: studies in which the economic evaluation was conducted as a secondary analysis.

+ ROBINS-I assessment skipped pre-assessment steps, as the EE deployed Markov models.

a Multiple sources, based on pooled analysis of multiple studies.

b Multiple sources, applied to multiple scenarios.

c Multiple sources.

d Multiple sources, including Medicare and Medicaid data.

e Multiple sources, including real-world data.

f Multiple sources, deploying retrospective and prospective data for training and validation.

*NAs were assigned to questions on modeling for model-based EEs only, such that a maximum of 70 points in CHEQUE was achievable in other EE types.

† Use of reporting guidelines.

## Discussion

This review identified substantial heterogeneity in the methods, reporting practices, and quality of EEs of DHTs. This general verdict aligns with other systematic reviews that focused on internet- and mobile-based interventions (33, 101). Across the included studies, three main findings emerged. First, most EEs relied on conventional approaches, particularly CUAs and RCTs, with limited adaptation to DHT-specific characteristics. Second, reporting and methodological quality were inconsistent, including incomplete descriptions of interventions, perspectives, costing methods, and handling of uncertainty. Third, key methodological gaps were identified, including insufficient consideration of uncertainty and heterogeneity, limited transparency in costing approaches, and a lack of attention to DHT-specific aspects such as technology life cycles and transferability.

While some heterogeneity might be explained by internal factors, such as different perspectives used, certain tendencies emerged, including the widespread reliance on RCTs. In particular, many RCT-based EEs were characterized by short time horizons and limited sample sizes, restricting their ability to detect meaningful differences in costs and outcomes and thereby potentially limiting real-world relevance (102, 103). These limitations were empirically observable in the included studies rather than being specific to DHTs *per se*. As such, the challenge lies less in the use of RCTs themselves and more in how they are implemented in the context of EEs. Furthermore, none of the included studies explicitly accounted for the rapid development cycles typical of DHTs, despite methodological approaches such as sensitivity analyses or iterative study designs potentially addressing this issue. Potential approaches discussed in the literature (18, 19) include adapting study designs and analyses to better reflect the evolving and context-dependent nature of DHTs, for example through sensitivity analyses or the use of additional data sources, although such approaches were rarely applied in the included studies identified in this review.

Both RCTs and non-RCTs face challenges related to the use of external digital interventions. Participants may access alternative DHTs outside of study protocols, which may influence outcomes and obscure intervention effects (18, 104). Although approaches such as exclusion criteria or statistical adjustments may mitigate this issue, these were not applied in the included studies, and only one study described the exclusion of participants using similar DHTs prior to study enrollment (82). More broadly, the use of external DHTs is closely linked to questions of usability and sustained engagement, which are critical for achieving robust intervention effects (108). The limited consideration of these aspects in the included EEs suggests that important determinants of real-world effectiveness, such as user behavior and adherence, remain insufficiently captured in the included studies.

CUA dominated the included evaluations, with QALYs used as the primary outcome in the majority of studies. Only EEs conducted as a secondary objective used CEA more frequently. However, this reliance on QALYs contrasts with the characteristics of many included studies, which often had short time horizons and limited statistical power. Although measurement of natural outcomes is useful, it limits generalizability in the context of CEA and highlights the multidimensionality of DHTs, which is a challenge to evidence generation and evaluation for policy decision. A related issue concerns the widespread reliance of QALYs as the sole primary outcome (23), despite the fact that RCTs are rarely powered to detect differences in QALYs or costs (101). For this reason, cost-consequence analyses (CCAs) have been recommended to capture multiple dimensions of value (19, 23). No CCAs were identified in this review, representing a missed opportunity, particularly when EEs can be planned and adequately powered alongside studies. This may be explained by the increased complexity of conducting CCA.

A key finding of this review was the limited transparency and consistency in costing approaches. Sample sizes were small overall, though larger than those reported in a previous SLR of telemedicine technologies (105). Excluding model-based EEs, only 9 EEs justified their sample size (e.g., with power analysis) (50, 52, 54, 58, 78, 79, 92, 94, 97), while others discussed limitations in powering outcomes or costs (55, 57, 70, 73). The validity and generalizability of long-term outcomes were limited by short time horizons, usually below one year, except in model-based EEs relying on multiple secondary sources (59–64, 71, 75, 76, 83–86, 90, 99). However, model-based EEs have their own limitations, including abstraction of modelling, potential misalignment with DHT life cycles, and complexity in managing heterogeneous information sources (106). Many interventions were not stand-alone but comprised components with complex systems, complicating attribution of effects of interventions to the DHT itself and carrying the difficulty of differentiation of effects from the central DHT (17, 18), particularly when auxiliary devices lacked a defined medical purpose (62, 74, 80, 96) or additional devices used in a real-world setting may differ from study devices in quality and type. Notably, none of the included studies explicitly accounted for technology life cycles or iterative updates, despite being frequently highlighted as key characteristics of DHTs in the literature (18, 19).

The societal perspective was the most frequently used (31% of EEs) and is generally recommended for capturing the multidimensional costs and benefits of DHTs (19). However, national guidances do not uniformly recommend a societal perspective. Several countries use narrower or decision-maker-oriented reference cases, such as the NHS (personal social services perspective) in England, the statutorily-insured perspective in Germany, the publicly funded health care payer perspective in Canada, and the collective or healthcare-system perspective in France (107–110). While a bottom-up-approach was commonly applied for valuing resources, a key finding of this review was the limited transparency and consistency in costing approaches. Substantial heterogeneity and frequent lack of reporting in valuation methods reduced comparability across EEs. In particular, the distinction between bottom-up and top-down approaches was often unclear, especially in model-based EEs. An emphasis on intervention costs was common, in contrast to the findings of other SLRs of internet- and mobile-based mental health interventions (101). These costs mainly comprised of licensing and device costs at the disadvantage of other cost categories and generalizability as suggested by other SLRs (111, 112). Where cost and resource use relied on self-reporting, studies were vulnerable to recall bias and social desirability bias (111). Similar heterogeneity was observed for the examination of uncertainty. Although most EEs performed sensitivity analyses, these often did not capture the full scope of uncertainty. Structural uncertainty was rarely addressed, and heterogeneity or distributional effects were only considered in a minority of studies. Only three model-based EEs discussed heterogeneity and distributional effects (60, 61, 84). Some degree of heterogeneity is inherent and expected across interventions, indications and contexts, but the observed gaps in reporting and methodological quality extend beyond this inherent variability.

During screening, we noted that digital interventions and study perspectives were sometimes poorly described, which hindered exact classification. Insufficient specification of the intervention and its intended mechanism of benefit impede transparency to patients, stakeholders, and researchers (18, 19). While some EEs used reporting guidelines such as the CHEERS, the findings highlight the need for adapted guidelines that better reflect DHT-specific reporting requirements, such as the AI-adapted CHEERS-(AI) (48). This is particularly important for ensuring consistent quality assessment and communication of the DHTs value and health-economic implications. Hence, underreported CHEERS-(AI) items concerned contextual information on settings, intervention strategies, sub-group impacts, and user, community, or stakeholder engagement. Primary studies and EEs were rarely pre-registered, and certain methodological details, such as modeling assumptions and statistical analysis, were often missing or insufficiently discussed, complicating methodological appraisal. Most prominently, CHEQUE(–Domain M) assessment revealed deficiencies in addressing uncertainty, model structure, and model validation. In model-based EEs, the multiplicity of sources and their justification for parameter selection further hindered reporting quality and RoB assessment. Similarly to CHEERS(-AI), this fed back into problems of relevant equity or distributional considerations. While model-based studies hence suffered from complexity issues, most non-randomized primary studies exhibited high RoB due to lack of adjustment, usually in ROBINS-I pre-assessment. Given the limitations of the currently available tools, the findings highlight the importance of distinguishing between limitations arising from underlying study designs and those related to economic evaluation methods. While different assessment tools address distinct aspects such as reporting quality and risk of bias, the findings suggest a need for better alignment and adaptation of existing frameworks to capture DHT-specific challenges. Existing approaches may benefit from improved consideration of DHT-relevant aspects, such as real-world relevance, stakeholder engagement, modeling assumptions, and distributional considerations, while maintaining the use of complementary tools addressing distinct domains.

A key strength of this review is the large number of studies included and the focus on DHTs according to the MDR definition. The density of information retrieved allowed insights into the methods across EE types. However, an important limitation lies in the broad inclusion of all types of DHTs and clinical indications, which constrained the depth of synthesis and critical appraisal of methodology. Similarly, the rapid review design is limited by a less comprehensive synthesis, limited database inclusion, and potential biases from accelerated screening compared to a systematic review (113). The literature search was limited to four years, two databases and English or German publications, potentially omitting relevant studies. Hence, the results of this rapid review may be blind to methodologies of studies published in other languages, which can reflect different national approaches with their strengths and weaknesses. The time window was chosen to represent an up-to-date landscape of studies performed and concluded given the recent uptake and significance of DHTs, especially after the Covid-19 pandemic (2, 3). A comprehensive set of quality assessment instruments was deployed to cover all aspects of included primary studies. However, model-based studies posed particular challenges due to their often-numerous data sources and the lack of DHT-specific assessment tools. Many included studies were also affected by incomplete reporting and missing information that influences both quality assessment and synthesis.

In summary, the findings underscore a fragmented landscape in which heterogeneity in EE designs, perspectives, costing methods and outcome measures impede comparability and broader policy applicability. Despite the growing importance of DHTs, many evaluations did not sufficiently address the inherent challenges or leverage the opportunities described in the literature. Consistent with concerns raised by previous literature (18, 102, 103), this review shows that the standard EE methodologies developed for non-digital interventions may be inappropriate to assess DHTs yet remain widely used. Key issues span three domains: 1. Transparent, comprehensive and clear reporting to allow for full appraisal of the benefits of DHTs. 2. Bias arising from underlying studies, especially when multiple sources not designed for EE of DHTs are involved, and 3. methodological limitations in accounting for uncertainty and potential biases arising from data and modeling choices. Collectively, these concerns suggest that EEs may overstate the relevance and scalability of DHTs for real-world use, constraining the realization of their potential public health benefits.

Building on the findings of this review, a clear but flexible regulatory framework is needed, including guidance on economic evidence generation. In addition to accommodating the specificities of diverse and continuously developing DHTs, foundational issues related to publication practices must also be addressed, such as adherence to reporting standards and routine pre-registration of studies. Future research should therefore focus on improving methodological transparency and adapting economic evaluation approaches to better reflect the dynamic and context-dependent nature of DHTs.
