# Supplementary material for: Health economic evaluations of digital health technologies—a rapid review of applied methods
Source: Front Digit Health. 2026 Jul 2;8:1816757. doi: 10.3389/fdgth.2026.1816757 (PMC13372764; doi:10.3389/fdgth.2026.1816757)
Supplement: Supplementary file 1 [file Datasheet1.pdf]

Appendix

Appendix 1: Search strings

**PubMed**	("digital health" [Mesh] OR „digital health" [tiab] OR "digital therapeutic*"[tiab] OR „telemedicine" [Mesh] OR „telemedicine"[tiab] OR „Wearable Electronic Devices"[Mesh] OR „digital technology"[Mesh] OR „remote consultation"[tiab] OR „home care services"[tiab] OR „telenursing"[tiab] OR „telemetry"[Mesh]) AND ("Cost-Benefit Analysis"[Mesh] OR "Cost-Effectiveness Analysis"[Mesh] OR "cost effectiveness"[tiab] OR "economic assessment"[tiab] OR "cost consequence"[tiab] OR "cost-consequence"[tiab] OR "hta"[tiab] OR "health technology assessment"[tiab])
**Scopus**	(TITLE-ABS ("digital health" OR "digital therapeutic*" OR "telemedicine" OR "virtual medicine" OR "tele-referral*" OR "mobile health" OR "mhealth" OR "telehealth" OR “tele-health” OR “tele health” OR "ehealth" OR "tele-intensive care" OR "tele-ICU" OR "telecare" OR "tele care" OR "tele-care" OR "distance counseling" OR "e-counseling" OR "e counseling" OR "e-therap*" OR "mental health teletherapy" OR "telepsychotherapy" OR "tele psychotherapy" OR "tele-psychotherapy" OR "telepsychology" OR "telepathology" OR "teleradiology" OR "telerehabilitation" OR "virtual rehabilitation" OR "tele-rehabilitation" OR "tele rehabilitation*" OR "remote rehabilitation" OR "remote consultation" OR "teleconsultation*" OR "tele consultation*" OR "tele-consultation*" OR "electronic health" OR "wearable electronic device" OR "wearable device*" OR "wearable technolog*" OR "electronic skin" OR "wearable computer*" OR "digital technolog*" OR "digital electronic*" OR "home care services" OR "telenursing" OR "tele nursing" OR "tele-nursing" OR "telemetry") AND TITLE-ABS ("cost-benefit" OR "cost benefit" OR "cost* and benefit*" OR "cost-utility" OR "cost utility" OR "marginal analysis" OR "economic evaluation*" OR "cost-effectiveness" OR "cost effectiveness" OR "cost-effectiveness-ratio" OR "economic assessment" OR "cost consequence" OR "cost-consequence" OR "hta" OR "health technology assessment")) AND PUBYEAR > 2013 AND PUBYEAR < 2025 AND (LIMIT-TO (DOCTYPE , "ar") OR LIMIT-TO (DOCTYPE , "re")) AND (LIMIT-TO (SUBJAREA , "MEDI") OR LIMIT-TO (SUBJAREA , "NURS") OR LIMIT-TO (SUBJAREA , "PHAR") OR LIMIT-TO (SUBJAREA , "HEAL")) AND (LIMIT-TO (LANGUAGE , "English") OR LIMIT-TO (LANGUAGE , "German"))

Appendix 2: Characteristics of economic evaluations as a primary objective

EE (first author)	Based on study type:	EE-Type[1]	Time horizon	Intervention aims and target population	Sample size	Description of intervention	Components of intervention	Description of comparator	Summary of results[1]
Ambrens 2022 (74)	RCT	CEA<br>CUA	2 Years	**Aim**: Decrease in falls and fall injuries<br>**Population**: English-speaking patients (≥ 70 years), independently living and able to leave the home without walking aid	503	The StandingTall program consists of tailored exercises delivered via tablet and additional devices, provided alongside usual care. Implemented in a home setting.	-App<br>-Additional aid material (e.g. foam cushion)	Health education via tablet computer, but without the StandingTall program.	CEA:<br>$+C^{n.s.}$ $+E^{s}$<br>CUA:<br>$+C^{n.s.}$ +E
van der Hout 2021 (51)	RCT	CUA	6 Months	**Aim**: Self-management of cancer survivors<br>**Population**: Survivors of cancer, (≥ 18 years) and 3 months up to 5 years after curative therapy of different cancers	625	"Oncokompas" supports users through self-monitoring of outcomes, tailored information, and personalized care options based on results. Used alongside standard therapy and implemented in a home setting.	-App<br>-Standard care	The comparison group was the usual treatment with a waiting list.	CUA:<br>$dominant^{s}$
Mujcic 2022 (50)	RCT (pragmatic)	CEA<br>CUA	1 Year	**Aim**: Management for survivors of cancer with abusive alcohol use<br>**Population**: Participants (≥ 18 years), with any diagnosis of cancer over the last 10 years and alcohol consumption above Dutch guideline recommendation	103	The digital intervention provides interactive exercises, a self-monitoring diary, and peer support to reduce alcohol consumption among cancer survivors, accessible via digital devices. Implemented in a home setting.	-App	A non-interactive brochure provides general information and tips for moderate alcohol consumption tailored to cancer survivors. It is available online without interactive features or reminder aids.	CEA:<br>$dominant^{n.s}$<br>CUA:<br>$-C^{n.r}$ $-E^{n.s}$
Lopez-Villegas 2020 (52)	RCT	CUA	1 Year	**Aim**: Surveillance of pacemakers<br>**Population**: Patients with single or two-chamber pacemaker	50	Pacemaker with enhanced capabilities and a CardioMessenger for wireless communication with the Biotronik Home Monitoring System, enabling encrypted transmission of health data to a Biotronik service center for analysis and monitoring. Implemented in a home setting.	-Remote monitoring system<br>-Pacemaker<br>-Additional devices	Patients with similar types of pacemakers, but who were managed through traditional hospital visits, with regular consultations required for device checks and clinical assessments.	CUA:<br>$+C^{n.s}$ $+E^{n.s}$ from two perspectives (health care system and societal)
Nelson 2021 (68)	RCT	CUA	6 Months	**Aim**: Rehabilitation after total hip arthroplasty<br>**Population**: Patients with total hip arthroplasty	70	Telerehabilitation via two applications on a tablet computer, including remote physiotherapy sessions and home-based exercises. Implemented in a home setting.	-Two Apps	Traditional in-person rehabilitation with standard home exercise programs and physiotherapy appointments.	CUA:<br>$dominant^{n.s}$
Buntrock 2022 (57)	RCT (pragmatic)	CEA<br>CUA<br>CBA	6 Month	**Aim:** Reduction of alcohol misuse<br>**Population:** Employees with problematic alcohol use	432	Guided and unguided web-based evidence based trainings for treatment of problematic alcohol use.	- Web-based application	Usual care (S3-Guideline for alcohol related disorders).	CEA/CUA/ CBA:<br>$dominant^{n.r}$
Dawkins 2024 (53)	RCT	CUA	Primary:<br>18 Weeks<br>Secondary:<br>1 Year	**Aim**: Symptomatic therapy while receiving chemotherapy<br>**Population**: Patients receiving systemic treatment for colorectal, breast or gynecological carcinoma	508	The eRAPID system is an online eHealth intervention that enables patients to self-report symptoms and side effects during chemotherapy, provides automated severity-based advice, and integrates self-reports into electronic health records. Implemented in a home setting.	-App	Usual care includes conventional symptom management without the use of the eR-APID system, typically through outpatient clinic visits and emergency care.	CUA:<br>$dominant^{n.s}$<br>(in both primary and secondary analysis)
Fatoye 2020 (54)	RCT	CUA	8 Weeks	**Aim**: Rehabilitation of unspecific chronic back pain<br>**Population**: Patients (20-65 years) with a diagnosis of unspecific chronic lower back pain	47	Telerehabilitation-based McKenzie Therapy (TBMT) is a mobile app for guided self-management based on the McKenzie Extension Protocol, including exercises and e-learning modules. Implemented in a home setting.	-App	Clinical McKenzie therapy (CBMT) includes in-person sessions using the McKenzie Extension Protocol along with back education.	CUA:<br>$dominant^{n.s}$

Pelle 2022 (69)	RCT	CEA<br>CUA	6 Months	**Aim**: Therapy of osteoarthritis of the knee or hips<br>**Population**: Adults (≥ 50) years with self-reported osteoarthritis symptoms in knee and/or hips	427	Dr. Bart supports self-management through goal setting for a healthier lifestyle, based on the Fogg Behavior Model, incorporating reminders, rewards, and self-monitoring. Used alongside standard therapy and implemented in a home setting.	-App<br>-Standard care	The comparison group is the usual care, consisting of standard treatments provided by healthcare professionals.	CEA: -C =E<br>CUA: dominant
Schuit 2022 (55)	RCT	CUA	3 Months	**Aim**: Self-management of uncurable cancers (various tumor types)<br>**Population** Adults (≥ 18 years) with uncurable cancer and life expectancy of at least three months	138	Oncokompas, a fully automated online application for self-management, providing symptom-tailored feedback and advice. Used alongside standard therapy and implemented in a home setting.	-App<br>-Standard care	Standard palliative care	CUA:<br>-$C^{n.s}$ -$E^{n.s}$
Bautista-Mesa 2020 (73)	Controlled clinical study	CUA	5 Years	**Aim**: Surveillance of the function of pacemakers<br>**Population**: Older patients with pacemakers	55	Remote monitoring via the Medtronic CareLink® Network for pacemaker function monitoring. Implemented in a home setting.	-Application devices (including software)	Conventional monitoring (CM) with scheduled hospital visits for pacemaker monitoring.	CUA:<br>+$C^{n.s}$ -$E^{n.s}$
Dagenais 2021 (91)	RCT	CEA<br>CUA<br>CBA	1 Year	**Aim**: Decrease of absences from work due to lower back pain<br>**Population**: Firefighters	216	Participants had access to an electronic portal with exercise videos, automated reminders, a progress tracking log, performance reports, and remote support via phone, email, or SMS.<br>Implemented in a home setting.	-App	Two comparison groups:<br>1:1 care; group course.	[2, n.r.]
de Jong 2020 (56)	RCT (pragmatic)	CUA	1 Year	**Aim**: Surveillance of inflammatory bowel disease<br>**Population**: Patients (18-75 years) with inflammatory bowel disease	909	myIBDcoach, a telemedicine tool for monitoring disease activity, medication adherence, nutrition, and psychosocial factors, with e-learning modules, personalized care plans, and more intensive monitoring during flare-ups.<br>Implemented in a home setting.	-App	Outpatient standard care, which includes regular, scheduled visits to healthcare providers without the use of telemedicine.	CUA:<br>$dominant^{n.s}$
Mudiyanse lage 2023 (70)	Pilot-RCT	CUA	1 Year	**Aim**: Management of chronic conditions<br>**Population**: Patients with a diagnosis of COPD and/or diabetes	172	Telemonitoring through diagnosis-specific devices (e.g., blood glucose meters), tablet computer with TELUS package (software for telemedicine home monitoring). Implemented in a home setting.	-App<br>-Additional devices	Usual care without telemedicine intervention.	CUA:<br>+$C^{n.s}$ +E^{s}
Boggs 2022 (78)	RCT	CEA	1 Year	**Aim**: Decrease or relapses in severe depressive symptoms<br>**Population**: Adults with at least one prior severe depressive episode and current residual symptoms	389	Mindful Mood Balance (MMB) is a web-based program that teaches mindfulness and meditation skills to reduce remaining depressive symptoms and prevent recurrences; used alongside usual depression treatment.<br>Used alongside standard therapy and implemented in a home setting.	-App<br>-Standard care	Standard therapy for depression, typically including clinical standard procedures for treating depression.	CEA:<br>+$C^{n.r}$ +$E^{n.r}$
Bernard 2022 (49)	RCT (prospective, pragmatic)	CUA	6 Months	**Aim**: Management/surveillance of patients with rheumatoid arthritis<br>**Population**: Patients with rheumatoid arthritis beginning DMARD therapy	89	SATIE-PR application for monitoring health status from home during DMARD therapy. Implemented in a home setting.	-App	Conventional monitoring with regular visits to a rheumatologist.	CUA: dominant ($E^{n.s}$)
Liu/Tang 2023 (89)	RCT	CUA	12 Weeks	**Aim**: Rehabilitation from atrial fibrillation<br>**Population**: Patients (18-75 years) with atrial fibrillation, undergoing catheter ablation	97	ShuKang application with personalized training guidelines, remote monitoring via wearable devices, real-time heart rate measurement, and training instructions. Implemented in a home setting.	-App<br>-Additional devices	Conventional home-based cardiac rehabilitation.	CUA:<br>+$C^{n.s}$ +E^{s}
Mourad 2022 (67)	RCT	CUA	1 Year	**Aim:** Improvement of mental health of patients with cardiovascular diseases through CBT<br>**Population**: Patients with cardiovascular diseases suffering from depressive symptoms	138	Internet-based cognitive behavioral therapy (iCBT) with goal setting, psychoeducation, problem-solving, behavioral activation, including homework with weekly written feedback. Implemented in a home setting.	-App	Online discussion forum (ODF) with moderated discussions on various topics.	CUA:<br>+$C^{n.s}$ +E^{s}

Lam 2024 (93)	RCT	CEA	6 Months	**Aim**: Therapy of survivors of stroke<br>**Population**: Adults at first or repeated ischemic/hemorrhagic stroke	256	The Virtual Multidisciplinary Stroke Care Clinic (VMSCC) includes virtual individual sessions with a nurse, home blood pressure telemonitoring, and unlimited access to an online resource platform.<br>Used alongside standard therapy and implemented in a home setting.	-App<br>-Blood pressure measuring device<br>-Standard care	Usual care alone, without the VMSCC service.	CEA:<br>$+C^{n.r}$ $+E^{n.r}$
Ney 2021 (77)	RCT	CUA	1 Year	**Aim**: Therapy of COPD<br>**Population**: Veterans in the US with COPD	238	Participants received a pedometer, uploaded their daily steps at least once a week, received personalized step goals, and gained access to a website with four key components: (1) individual goal setting, (2) iterative feedback, (3) motivation and educational content, and (4) an online community forum.<br>Used alongside standard therapy and implemented in a home setting.	-Web-app<br>-Activity monitor	Two comparison groups:<br>I: Pedometer without step count goals or instructions to increase physical activity.<br>II: Written prescription to increase physical activity and encouragement to exercise, unsupervised.	CUA IVG I:<br>$+C^{n.r}$ $+E^{n.s}$<br><br>CUA IVG II:<br>$+C^{n.r}$ $+E^{n.s}$
Lemelin 2020 (92)	Prospective, controlled clinical non-inferiority study	CEA	NA	**Aim**: Patient management for gestational diabetes<br>**Population**: Pregnant women with diagnosed gestational diabetes	161	Telehomecare system for remote monitoring and control of blood glucose levels, including the transmission and analysis of capillary glucose data. Access to an online resource platform; used alongside usual care and implemented in a home setting.	-Web-app<br>-Glucose measuring device	The comparison group consisted of usual care in the clinic, which included standard in-person follow-up and medical visits for the treatment of gestational diabetes.	CEA:<br>dominant[2]

[1]C: Costs | E: Effect on outcomes | +: increased costs or outcomes | -: decreased costs or outcomes | =: constant/equal cost or outcomes | NA = Not available | IVG I: Intervention group 1 | IVG II: Intervention group 2
[2]versus C1: CEA: -K +E / CUA: -K -E / CBA: | versus C2: CEA: +K +E / CUA: +K -E / CBA: - | EEs as a primary objective: studies in which the economic evaluation was the primary objective
^{s}: significant | $^{n.s}$: not significant | $^{n.r}$: not reported | NA: Not applicable | CEA: Cost-effectiveness analysis | CUA: Cost-utility analysis | CBA: Cost-benefit analysis

Appendix 3: Characteristics of economic evaluations as a secondary objective

EE (first author)	Based on study type:	EE-Type[2]	Time horizon	Intervention aims and target population	Sample size	Description of intervention	Components of intervention	Description of comparator	Summary of results[1]
Taylor 2020 (80)	RCT within an HTA	CEA<br>CUA	1 Year	**Aim**: Therapy of chronic conditions<br>**Population**: Participants (16-74 years) with a BMI of 30-40 kg/m^2 with hypertension, pre-diabetes, type-2-diabetes, Osteoarthritis of lower extremities or another prior history of therapy for depression	450	The e-coachER digital intervention offers web-based support for self-management, including a pedometer, daily physical activity logs, self-monitoring tools, and goal-setting features, delivered alongside usual care. It is accessible via digital devices and implemented in a home setting.	-App<br>-Additional devices (Pedometer)<br>-Printed materials	Standard exercise program	CEA:<br>$+C^{n.s}$ $+E^{s}$<br>CUA:<br>$+C^{n.s}$ $+E^{s}$
Sten-Gahmberg 2024 (58)	RCT	CBA	1 Year	**Aim**: Surveillance of chronic conditions<br>**Population**: Patients (≥ 18 years) with chronic conditions, significant burden of disease, medium to high risk of worsening of conditions and high health care utilization	730	Telemedical follow-up using tablets and home monitoring devices enables real-time data transmission to a care center, where nurses and general practitioners apply a traffic-light system for individualized support. Implemented in a home setting.	-App<br>-Additional devices	Usual care primarily led by the patient's general practitioner, tailored to their chronic conditions.	CBA: negative social value ($C^{n.r}$ E^{s})
Priebe 2024 (79)	RCT	CEA	1 Year	**Aim**: Therapy of lower back pain<br>**Population**: Patients (≥ 18 years) with chronic conditions, significant burden of disease, medium to high risk of worsening of conditions and high health care utilization	1,237	The Rise-up intervention includes access to the Kaia back pain app (exercise, mindfulness, education), supported by a shared electronic case report form (eCRF) and optional teleconsultations with pain specialists. Implemented in a home setting.	-App (Kaia Health App, eCRF)	Usual care provided by general practitioners following standard guidelines.	CUA: dominant^{s}
De Batlle 2021 (95)	Implementation trial	CEA	6 Months	**Aim**: Therapy of patients with complex chronic conditions<br>**Population**: Patients (≥ 55 years) with conic conditions and prior history of hospitalizations, living at home	76	An integrated care model combining a self-management app, a suite of connected sensors, and a web-based platform that links healthcare professionals to support patients and enhance care coordination. Implemented in a home setting.	-Two programs (App and platform)<br>-Additional devices	Usual care provided by primary care without the integrated mHealth tools.	CEA: dominant $^{n.s}$
Colomina 2021 (96)	Implementation trial	CEA	6 Months	**Aim**: Therapy of patients with complex, chronic osteoarthritis<br>**Population** Patients (> 65 years) with hip or knee prosthesis	59	The CONNECARE integrated care model included a self-management app with status and performance reports, a virtual coach, a digital activity tracker, a patient profile on a web-based care coordination platform, and support from a case manager. Implemented in a home setting.	-Two programs (App and platform)<br>-Additional devices	Usual care provided by primary care following hospital discharge, without the additional support of integrated care.	CEA: dominant ($C^{n.s}$ $+E^{s}$)
McManus 2021 (97)	RCT	CEA	1 Year	**Aim**: Management and surveillance of hypertension<br>**Population**: Adults with treated, but poorly controlled hypertension	622	HOME BP provided patients and healthcare professionals with feedback on blood pressure readings, along with optional lifestyle advice and motivational support. Implemented in a home setting.	-App (not clearly described)<br>-Additional devices	Usual care involving routine hypertension management, including appointments and medication adjustments at the discretion of the general practitioner.	CEA:<br>$+C^{n.r}$<br>$+E^{n.s}$
Smak-Gregoor 2023 (98)	Retrospective population-based study (pragmatic)	CEA	1 Year	**Aim**: Detection of skin cancer<br>**Population**: Health adults without prior history of skin cancer	78,840	SkinVision uses artificial intelligence to assess skin cancer risk based on photographs of skin lesions. Implemented in a home setting.	-App	The comparison group receives standard care, consisting of routine dermatological	CEA:<br>$+C^{n.r}$$+E^{s}$ (not clearly reported)

								examinations without the use of the mHealth app.	
Park 2023 (94)	Randomized experimental design	CUA	4 Weeks	**Aim**: Therapy of lower back pain **Population**: Random sample of 100 participants with chronic back pain (mean age 35,5 ± 8,8 Jahre; 40 female patients)	100	Dr. AI is a digital app-based physiotherapy program that uses a deep learning system to primarily deliver exercise-based interventions. Implemented in a home setting.	-App	Conventional physiotherapy (CPT) involving in-person treatment sessions.	CUA: dominant ($C^{n.r}$ E^{s})

[1]C: Costs | E: Effect on outcomes | +: increased costs or outcomes | -: decreased costs or outcomes | =: constant/equal cost or outcomes

^{s}: significant | $^{n.s}$: not significant | $^{n.r}$: not reported | NA: Not applicable | CEA: Cost-effectiveness analysis | CUA: Cost-utility analysis | CBA: Cost-benefit | EEs as a secondary objective: studies in which the economic evaluation was conducted as a secondary analysis

Appendix 4: Characteristics of model-based economic evaluations

EE (first author)	Based on study type:	EE-Type[3]	Time horizon	Intervention aims and target population	Sample size	Description of intervention	Components of intervention	Description of comparator	Summary of results[1]
Greenwood 2024 (82)	RCT with model-based EE	CUA	6 Months	**Aim**: Improvement of mental health with CKD through physical activity<br>**Population:** Adults with CKD	340	The Kidney-BEAM program offers structured digital exercises, live sessions, and an on-demand pre-recorded rehabilitation program. Implemented in a home-based setting.	-App	Waitlist control group (referred to the Kidney-BEAM platform after the 12-week assessment).	CUA: $+C^{n.r}$ $+E^{s}$ (Base Case and Complete Case)
Bhardwaj 2021 (72)	Prospective study	CUA	1 Year	**Aim**: Rehabilitation after AMI<br>**Population**: Adult patients hospitalized for myocardial infarction	1,064 (200/864 historical control group)	Corrie is a smartphone-based care model that combines a mobile app, smartwatch, and wireless blood pressure monitor to support medication tracking, education, vital sign monitoring, and care coordination. Implemented in a home setting.	-App<br>-Additional devices<br>-Standard care	Usual care alone, typically consisting of standard treatment approaches for AMI patients following discharge.	CUA: dominant $^{n.r}$
Caillon 2022 (60)	Diverse studies	CUA	10 Years	**Aim:** Monitoring following acute exacerbation of heart failure<br>**Population:** Patients, primarily post-hospitalization due to an acute worsening of heart failure	10,000 (hypothetical)	The SCAD program is an interactive telemonitoring service that allows patients to enter data on their clinical status, lifestyle, mental health, and treatment adherence, which is monitored by healthcare professionals. Implemented in a home setting.	-App	Usual in-hospital care for patients with heart failure without telemonitoring.	CUA: $+C^{n.r}$ $+E^{s}$
Davison 2024 (61)	RCT + Diverse studies	CUA	Lifetime	**Aim:** Cognitive Behavioral Therapy for type 2 diabetes<br>**Population:** Adults with T2D	610	BT-001 (AspyreRx™) targets behaviors related to blood glucose control in patients under medical care and can be used as an adjunct to pharmacological diabetes treatments, in addition to usual care. Implemented in a home setting.	-App<br>-Standard care	Usual care, including health education on diet, physical activity, and weight loss, as well as the composition of oral and injectable treatments for type 2 diabetes.	CUA: $+C^{n.r}$ $+E^{n.r}$
Freitag 2024 (83)	Diverse studies	CUA	5 Years	**Aim:** Treatment of depression<br>**Population:** Adults with depression in Germany	4,977,000	DiGA offering cognitive behavioral therapy exercises, mood assessments, and psychoeducation to improve access to mental health care. Implemented in a home setting.	-App	The comparison group received standard treatment for patients with depression without the use of DiGA.	CUA: $+C^{s/\ n.s}$ $+E^{n.r}$ (in two scenarios)
Park 2024 (62)	Diverse studies	CUA	10 Years	**Aim:** Diabetes prevention<br>**Population:** Patients with an HbA1c level of 5.89%	NR	The digital Diabetes Prevention Program (d-DPP) provides access to a range of health-promoting resources, including coaching by a lifestyle health coach, discussions with a virtual peer group, meal tracking, and weight monitoring via a wearable device. Implemented in a home setting.	-Multiple apps<br>-Wearables	In-person DPP, a lifestyle intervention program focused on achieving and maintaining weight loss, accompanied by a 16-session core curriculum on behavior change.	CUA: dominant (from two perspectives) $^{n.r}$
Lin 2023 (64)	Diverse studies	CEA; CUA	30 Years	**Aim:** Screening for diabetic retinopathy<br>**Population:** Community-dwelling individuals with diabetes (Shanghai)	100,000 (hypothetical)	AI-assisted screening for diabetic retinopathy. Implemented in a clinical setting.	-App	Telemedicine-based screening for diabetic retinopathy using manual grading.	CEA: dominant $^{n.r}$<br>CUA: dominant $^{n.r}$
Lewkowicz 2023 (65)	RCT + Diverse studies	CUA	3 Years	**Aim:** Treatment of low back pain<br>**Population:** Individuals with low back pain (no further details; hypothetical cohort)	10,000 (hypothetical)	Kaia Health app delivers unsupervised treatment through video exercises and educational materials. Implemented in a home setting.	-App	Treatment as usual, including in-person physiotherapy and pain relief medication.	CUA: $+C^{n.r}$ $+E^{n.s}$

Morrison 2022 (76)	Diverse studies	CUA	Life time	**Aim:** Early screening for retinopathy<br>**Population:** Infants born at 30 weeks' gestation or earlier or weighed 1500g or less at birth	52,000 (hypothetical)	Deep learning–based artificial intelligence (AI) software for autonomous and assistive screening of retinopathy of prematurity (ROP); used autonomous and assistive.	-App	Standard ophthalmologic face-to-face examination; telemedicine Telemedicine-based retinal image evaluation by ophthalmologist	CUA:<br>Autonomous AI dominant over both comparators $^{n.r}$ (in two scenarios)<br><br>Assistive AI dominant over ophthalmoscopy; $+C^{n.r}$ $+E^{n.r}$ compared to telemedicine (in two scenarios)
Nomura 2022 (84)	RCT	CUA	Lifetime (50 Years)	**Aim:** Treatment of hypertension<br>**Population:** Individuals with hypertension not receiving antihypertensive medication	1,000,000 iterations<br>-Based on study data of 390 patients	Prescription digital therapeutics (DTx) for hypertension, specifically the HERB Mobile System, which includes a smartphone app for patients and web-based management software for clinicians, combined with guideline-based lifestyle modification counseling, in addition to usual care. Implemented in a home setting.	-App<br>-Web-Tool<br>-Standard care	Usual care including standard lifestyle modification counseling without the use of the DTx system.	CUA:<br>$+C^{n.r}$ $+E^{n.r}$
Piera-Jiménez 2020 (66)	Cohort study	CUA	8 Months and 40 years (Model)	**Aim:** Integrated care for chronic conditions<br>**Population:** Patients (≥65 years) with special health needs due to chronic diseases and requiring social support	-No direct participants<br>- Model based partly on study with 198 patients	Telemedicine platform (Health Insight Solutions Homecare Platform) featuring safety sensors, medical devices, games, a personal diary, and a video conferencing system, in addition to standard care. Implemented in a home setting.	-App<br>-Additional devices (e.g. scale, blood sugar measuring device)<br>-Additional to standard care	Participants received integrated health and social care supported by a proprietary Enterprise Resource Planner (ERP).	CUA:<br>$+C^{n.r}$ $+E^{n.r}$
Senanayake 2023 (85)	RCT + Diverse studies	CUA	5 Years	**Aim:** Rehabilitation for CHD<br>**Population:** Patients with coronary heart disease or chronic heart failure (NYHA classes I, II, and III)	-No direct participants<br>-Model based partly on study with 198 patients	A 24-week hybrid cardiac telerehabilitation program combining 6 weeks of center-based sessions with 18 weeks of telerehabilitation, including tele-coaching, accelerometer-based activity monitoring, and online feedback, in addition to standard therapy. Implemented in a home setting.	-App<br>-Additional surveillance devices<br>-Standard care	Usual center-based cardiac rehabilitation (10 sessions over several weeks)	CUA:<br>$+C^{n.s}$ $+E^{n.s}$
Lin 2024 (63)	Diverse studies	CEA; CUA	30 Years	**Aim:** Screening for eye diseases<br>**Population:** Adults with diabetic retinopathy, age-related macular degeneration, glaucoma, or pathologic myopia	10,000 (hypothetical)	B: AI-assisted screening<br>C: AI-assisted screening using an alternative method.<br>Implemented in a clinical setting.	-App	Centralized screening using telemedicine systems based on manual grading.	CEA:<br>$+C^{n.r}$ $+E^{n.r}$<br>CUA:<br>$+C^{n.r}$ $+E^{n.r}$
Luo 2022 (71)	RCT + Diverse studies	CUA	30 Years	**Aim:** Integrated care for atrial fibrillation<br>**Population:** Patients with AF	-Not explicitly reported<br>-Model based partly on study with 2473 Patients	mHealth-based care integrating the ABC pathway through monitoring of various data and lifestyle recommendations. Implemented in a home setting.	-App<br>-Additional devices (pho-toplethysmo-graphy smart device)	The comparison group received usual care for patients with atrial fibrillation, following standard treatment guidelines without support from mHealth technology.	CUA:<br>$+C^{n.r}$ $+E^{n.r}$
Miranda 2022 (86)	RCT	CUA	1,25 Years	**Aim:** Self-management of chronic conditions<br>**Population:** Patients aged 60 years or older with complex chronic conditions	45	The ePRO tool consists of two main functions: first, *My Goal* for creating goal-oriented care plans; second, outcome measurement using validated questionnaires. Implemented in a home setting.	-App	Multidisciplinary primary care delivered by Family Health Teams.	CUA:<br>$+C^{n.r}$ $-E^{n.s}$
Patel 2020 (75)	RCT	CEA	5 Years	**Aim:** Decision aid for cardiovascular disease (CVD)<br>**Population:** Adults aged 45 years or older with a high risk of CVD	NR	HealthTracker is integrated into the electronic health record (EHR) to provide real-time decision support based on evidence-based national guidelines, a patient risk communication interface,	-App integrated into EHR-system	The comparison group received usual care without support from the HealthTracker tool.	CEA:<br>$+C^{n.r}$ $+E^{n.r}$

						and automated clinical auditing. Implemented in a clinical setting.			
Velez and Malone 2021 (87)	RCT + Diverse studies	CUA	12 Weeks	**Aim:** Treatment of opioid use disorder (OUD) **Population:** Individuals diagnosed with OUD, with a focus on treatment retention and effectiveness	NR	reSET-O is a prescription digital therapeutic that combines cognitive behavioral therapy with usual treatment. In addition to standard therapy. Implemented in a home setting.	-App -Standard care	Treatment as usual, including oral buprenorphine, in-person counseling, and contingency management.	CUA: dominant$^{n.r}$
Liu/Zhan 2023 (90)	RCT	CUA	10 Years	**Aim:** Rehabilitation for chronic heart failure (HF) **Population:** Patients with chronic HF across NYHA functional classes I–IV	2,315	ShuKang app provides exercises, monitoring of physiological indicators, and video instructions, with parameters measured by wearable devices. Implemented in a home setting.	-App -Additional device (Heart rate band)	Conventional home-based cardiac rehabilitation.	CUA: +C$^{n.r}$ +E^{s}
Velez 2021 (88)	RCT + Diverse studies	CUA	12 Weeks	**Aim:** Treatment of opioid use disorder (OUD) **Population:** Patients diagnosed with opioid use disorder	NR	reSET-O is a prescription digital therapeutic that combines cognitive behavioral therapy with usual care. In addition to standard therapy. Implemented in a home setting.	-App -Standard care	Treatment as usual, consisting of buprenorphine, in-person counseling, and contingency management.	CUA: dominant$^{n.r}$
Wang 2021 (81)	RCT + Diverse studies	CEA	12 Weeks	**Aim:** Treatment of opioid use disorder (OUD) **Population:** Adult patients with at least one diagnosis of OUD receiving treatment with buprenorphine	9,850	reSET-O is a prescription digital therapeutic that delivers cognitive behavioral therapy in combination with usual care. In addition to standard therapy. Implemented in a home setting.	-App -Standard care	Treatment as usual, consisting of buprenorphine, in-person counseling, and contingency management.	CEA: +C$^{n.r}$ +E$^{n.r}$
Zhang 2023 (59)	Cohort study	CUA	3 and 5 Years	**Aim:** Screening for retinoblastoma **Population:** Clinically suspected and treated patients with retinoblastoma	103	Deep learning algorithm (DLA-RB) for screening and monitoring of retinoblastoma. Implemented in a clinical setting.	-App	Standard ophthalmologic examinations.	CUA: dominant$^{n.r}$
Orchard 2020 (99)	Cross-sectional study	CEA; CUA	10 Years	**Aim:** Screening for atrial fibrillation (AF) **Population:** Patients aged 65 years and older without a prior diagnosis of AF	3,103	The intervention involved screening eligible patients for atrial fibrillation using smartphone ECG technology, supported by eHealth tools in clinical practices. Implemented in a clinical setting.	-App -eHealth-tools for clinics	No Screening.	No information on incremental costs or effects, reported only ICER/ICUR$^{n.r}$

[1]C: Costs | E: Effect on outcomes | +: increased costs or outcomes | -: decreased costs or outcomes | =: constant/equal cost or outcomes
^{s}:significant | $^{n.s}$: not significant | $^{n.r}$:not reported | NA: Not applicable | CEA: Cost-effectiveness analysis | CUA: Cost-utility analysis | CBA: Cost-benefit

Appendix 5: Overview of assessment of economic evaluation quality

Assessment of primary study quality: RoB2

**Stratum**	**EE first author**	**RoB2**					
		RoB due to randomization process	RoB due to deviations from intended interventions	RoB due to missing outcome data	RoB due to measurement of the outcome	RoB in selection of reported result	**RoB Overall**
**EEs as a primary objective (19/21)**	Ambrens 2022 (74)	Some concerns	Some concern	Low	Low	Some concern	Some concern
	Bernard 2022 (49)	Low	Low	Low	Low	Low	Low risk
	Boggs 2022 (78)	Low	Some concerns	Some concern	Some concern	Some concern	Some concern
	Buntrock 2021(57)	Some concern	Some concern	Low	Low	Some concern	Some concern
	Dagenais 2021 (91)	Low	Some concern	Some concern	Low	Some concern	Some concern
	Dawkins 2024 (53)	Low	High	Low	Some concern	Low	High risk
	de Jong 2020 (56)	Low	Some concern	Some concern	Low	Some concern	Some concern
	Fatoye 2020 (54)	Low	High	High	Low	Some concern	High risk
	van der Hout 2021 (51)	Low	Some concern	Some concern	Some concern	Some concern	Some concern
	Lam 2024 (93)	Some concerns	Low	Low	Some concern	Some concern	Some concern
	Liu/Tang 2023 (89)	Low	Some concern	Low	Some concern	Some concern	Some concern
	Lopez-Villegas 2020 (52)	Low	Some concern	Low	Some concern	Some concern	Some concern
	Mourad 2022 (67)	Low	Some concern	High	Low	Some concern	High risk
	Mudiyanselage 2023 (70)	Some concern	Some concern	Some concern	Some concern	Low	Some concern
	Mujcic 2022 (50)	High risk	Some concern	Low	Some concern	Some concern	High Risk
	Nelson 2021 (68)	Low	Low	Low	Some concern	Some concern	Some concern
	Ney 2021 (77)	Low	Low	Low	Some concern	Some concern	Some concern
	Pelle 2022 (69)	Low	High	Some concern	Low	Some concern	High Risk
	Schuit 2022 (55)	Low	Some concern	Some concern	Some concern	Some concern	Some concern
**EEs as a secondary objective (5/8)**	Taylor 2020 (80)	Low	Some concern	Low	Low	Low	Some concern
	Priebe 2024 (79)	Some concern	High	High	Some concern	Some concern	High risk
	McManus 2021 (97)	Some concern	High	Low	Low	Low	High risk
	Sten-Gahmberg 2024 (58)	Some concern	Some concern	Some concern	High	Some concern	High risk
	Park 2023 (94)	Low	High	High	Low	Some concern	High risk
**Model-based EEs (22)**	Bhardwaj 2021 (72)						
	Caillon 2022[a] (60)						
	Davison 2024 (61)	Low	Some concern	High	Some concern	Some concern	High risk
	Freitag 2024[b] (83)						
	Greenwood 2024 (82)	Low	Some concern	Low	Low	Low	Some concern
	Lewkowicz 2023 (65)	Low	High	High	Some concern	Some concern	High risk
	Liu/Zhan 2023 (90)	Low	High	High	Some concern	Some concern	High risk
	Lin 2024[c] (63)						
	Lin 2023[c] (64)						
	Luo 2022 (71)	Low	Some concern	High	Some concern	Some concern	High risk
	Miranda 2022 (86)	Low	High	High	Some concern	Some concern	High risk
	Nomura 2022 (84)	Low	Some concern	Some concern	Some concern	Some concern	Some concern
	Patel 2020 (75)	Low	Some concern	High	Some concern	Some concern	High risk
	Park 2024[d] (62)						
	Piera-Jiménez 2020 (66)						
	Senanayake 2023 (85)	Low	Some concern	Low	Low	Some concern	Some concern
	Velez and Malone 2021 (87)	Some concern	High	Low	Some concern	Some concern	High risk
	Velez 2021 (88)	Some concern	High	Low	Some concern	Some concern	High risk
	Wang 2021 (81)	Some concern	High	Low	Some concern	Some concern	High risk

	Zhang 2023[c] (59)						

NA: Not applicable

Note: If the study used multiple sources (especially for model-based EEs), the primary RCT was assessed if disclosed as such | [+]: ROBINS-I assessment skipped pre-assessment steps, as the EE deployed Markov-models | [a] Multiple sources | [b] Multiple sources, applied to multiple scenarios | [c] Multiple sources, including real-world data | [d] Multiple sources, based on pooled analysis of multiple studies | [e] Multiple sources, deploying retrospective and prospective data for training and validation | [†]: Use of reporting guidelines | EEs as a primary objective: studies in which the economic evaluation was the primary objective | EEs as a secondary objective: studies in which the economic evaluation was conducted as a secondary analysis

Assessment of primary study quality: ROBINS-I

Stratum	EE first author	ROBINS-I Domains								ROBINS-I
		Pre-assessment	Domain 1	Domain 2	Domain 3	Domain 4	Domain 5	Domain 6	Domain 7	
EEs as a primary objective	Bautista-Mesa 2020 (73)	X								Critical risk of bias
	Lemelin 2020 (92)	X								Critical risk of bias
EEs as a secondary objective	Colomina 2021 (96)	X								Critical risk of bias
	De Batlle 2021 (95)		Serious RoB	Critical RoB	Moderate RoB	Low RoB	Low RoB	Low RoB	Low RoB	Critical risk of bias
	Smak-Gregoor 2023 (98)		Moderate RoB	Moderate RoB	Low RoB	Low RoB	Low RoB	Low RoB	Low RoB	Moderate risk of bias
Model-based EEs	Bhardwaj 2021 (72)		Serious RoB	Serious RoB	Moderate RoB	Low RoB	Critical RoB	Low RoB	Low RoB	Critical risk of bias[+]
	Morrison 2022 [f] (76)									
	Orchard 2020 (99)	X								Critical risk of bias
	Piera-Jiménez 2020 (66)	X								Critical risk of bias

X: High RoB in pre-assessment | [+]: ROBINS-I assessment skipped pre-assessment steps, as the EE deployed Markov-models | [f]: Multiple sources, including Medicare and Medicaid data | EEs as a primary objective: studies in which the economic evaluation was the primary objective | EEs as a secondary objective: studies in which the economic evaluation was conducted as a secondary analysis

Assessment of methodological quality: CHEQUE, Domain M

Stratum	EE first author	CHEQE (Domain M)																								
		M1	M2	M3	M4	M5	M6	M7	M8	M9	M10	M11	M12	M13	M14	M15	M16	M17	M18	M19	M20	M21	M22	M23	M24	Out of 100 points (NAs counted as 0 points)
EEs as a primary objective (21)	Ambrens 2022 (74)	Yes	Yes	Partial	Yes	Partial	Yes	Yes	Yes	Yes	NA	NA	NA	NA	NA	NA	Yes	Partial	No	Yes	Yes	Yes	No	Partial	Partial	52.5
	Bautista-Mesa 2020 (73)	Yes	Yes	Yes	Yes	Yes	Yes	Yes	Yes	Yes	No	No	No	No	No	Partial	Yes	No	No	No	Yes	Yes	Yes	No	No	53.5
	Bernard 2022 (49)	Yes	Yes	Yes	Yes	Yes	Yes	Yes	Yes	NA	NA	NA	NA	NA	NA	NA	Partial	Yes	Yes	Yes	Yes	Yes	Yes	No	No	51
	Boggs 2022 (78)	Yes	Yes	Yes	Yes	Partial	No	Yes	Yes	NA	NA	NA	NA	NA	NA	NA	Yes	Yes	No	Yes	Yes	Yes	No	No	Partial	46
	Buntrock 2021(57)	Yes	Yes	Yes	Yes	Yes	Yes	Partial	Yes	NA	NA	NA	NA	NA	NA	NA	Partial	Yes	No	Yes	Yes	Yes	No	Yes	No	48
	Dagenais 2021 (91)	Yes	Partial	Yes	Yes	Yes	Yes	Yes	Yes	NA	NA	NA	NA	NA	NA	NA	Yes	Partial	No	Yes	Yes	Yes	Yes	No	Yes	47.5

	Dawkins 2024 (53)	Yes	Yes	Yes	Yes	Yes	Yes	Partial	Yes	Partial	NA	NA	NA	NA	NA	NA	Yes	No	No	Yes	Yes	Yes	Yes	No	No	48
	de Jong 2020 (56)	Yes	Yes	Yes	Yes	Yes	Yes	Yes	Yes	NA	NA	NA	NA	NA	NA	NA	Yes	No	Yes	Yes	Yes	Yes	Yes	No	No	49
	Fatoye 2020 (54)	Yes	Yes	Yes	Yes	Yes	Yes	No	Yes	NA	NA	NA	NA	NA	NA	NA	Partial	Partial	No	Yes	Yes	Yes	Yes	No	Yes	43.5
	van der Hout 2021 (51)	Yes	Yes	Yes	Yes	Yes	Yes	Yes	Yes	NA	NA	NA	NA	NA	NA	NA	Yes	Partial	Yes	Yes	Yes	Yes	Yes	No	No	51.5
	Lam 2024 (93)	Yes	Yes	Yes	No	Yes	No	Yes	Yes	NA	NA	NA	NA	NA	NA	NA	Partial	Partial	No	Yes	Yes	Yes	Yes	Yes	No	43.5
	Lemelin 2020 (92)	Yes	Yes	Yes	No	Yes	No	No	No	NA	NA	NA	NA	NA	NA	NA	Partial	Partial	NA	No	No	No	No	No	No	21.5
	Liu/Tang 2023 (89)	Yes	Yes	Yes	Yes	Yes	Yes	Partial	Yes	NA	NA	NA	NA	NA	NA	NA	Partial	No	Yes	Yes	Yes	Yes	Yes	Partial	Yes	47
	Lopez-Villegas 2020 (52)	Yes	Yes	Yes	Yes	Yes	Yes	Yes	Yes	NA	NA	NA	NA	NA	NA	NA	Yes	Yes	Yes	Yes	Yes	Yes	Yes	No	No	54
	Mourad 2022 (67)	Yes	Yes	Partial	Yes	Yes	Yes	Yes	Yes	NA	NA	NA	NA	NA	NA	NA	Partial	Yes	Yes	Yes	Yes	Yes	Yes	No	No	49
	Mudiyanselage 2023 (70)	Yes	Yes	Yes	Yes	Yes	Yes	Yes	Yes	NA	NA	NA	NA	NA	NA	NA	Yes	No	Yes	Yes	Yes	Yes	Yes	No	No	49
	Mujcic 2022 (50)	Yes	Yes	Partial	Yes	Partial	Yes	Yes	Yes	Yes	NA	NA	NA	NA	NA	NA	Yes	Yes	Yes	Yes	Yes	Yes	Yes	Partial	Partial	60
	Nelson 2021 (68)	Yes	Yes	Yes	Yes	Yes	Yes	Partial	Yes	NA	NA	NA	NA	NA	NA	NA	Yes	Yes	Yes	Yes	Yes	Yes	Yes	No	No	52
	Ney 2021 (77)	Yes	Yes	Yes	Yes	No	Yes	Yes	Yes	NA	NA	NA	NA	NA	NA	NA	No	Partial	No	Yes	Yes	Yes	Yes	Partial	No	44.5
	Pelle 2022 (69)	Yes	Yes	Yes	Yes	Yes	Yes	Yes	Yes	Yes	NA	NA	NA	NA	NA	NA	Yes	No	Yes	Yes	Yes	Yes	Yes	No	No	55
	Schuit 2022 (55)	Yes	Yes	Yes	Yes	Yes	Yes	Partial	Yes	NA	NA	NA	NA	NA	NA	NA	Yes	Partial	Yes	Yes	Yes	Yes	Yes	Partial	Yes	52.5
**EEs as a secondary objective (8)**	Colomina 2021 (96)	Yes	Yes	Yes	No	Yes	No	Yes	Yes	NA	NA	NA	NA	NA	NA	NA	Yes	No	NA	Yes	Yes	Yes	No	Yes	No	41
	De Batlle 2021 (95)	Yes	Yes	Yes	No	Yes	No	Yes	Yes	NA	NA	NA	NA	NA	NA	NA	Partial	Partial	NA	Yes	Yes	Yes	No	Yes	No	40.5
	McManus 2021 (97)	Yes	Yes	Yes	No	Yes	No	Yes	Yes	NA	NA	NA	NA	NA	NA	NA	Yes	Yes	No	Yes	Yes	Yes	Yes	No	Yes	46
	Park 2023 (94)	Partial	Yes	Yes	No	No	Yes	No	No	NA	NA	NA	NA	NA	NA	NA	Partial	Partial	No	Yes	Yes	Yes	Yes	No	Partial	31
	Priebe 2024 (79)	Yes	Yes	Yes	Yes	Yes	No	Yes	Yes	Yes	NA	NA	NA	NA	NA	NA	Yes	Yes	No	Yes	Yes	Yes	Yes	No	Partial	55.5
	Smak-Gregoor 2023 (98)	Yes	Yes	Yes	Yes	Yes	No	No	Yes	NA	NA	NA	NA	NA	NA	NA	Yes	Yes	NA	No	Yes	Yes	No	Yes	No	44
	Sten-Gahmberg 2024 (58)	Yes	Yes	Yes	Yes	Yes	Yes	Yes	Yes	NA	NA	NA	NA	NA	NA	NA	Yes	Yes	No	Yes	No	No	No	No	Partial	42.5
	Taylor 2020 (80)	Yes	Yes	Yes	Yes	Yes	Yes	Yes	Yes	Yes	NA	NA	NA	NA	NA	NA	Partial	Yes	Yes	Yes	Yes	Yes	Yes	No	Yes	58
**Model-based EEs (22)**	Bhardwaj 2021 (72)	Yes	Yes	Yes	Yes	Yes	Yes	Yes	Yes	Yes	Yes	Yes	Yes	No	No	Yes	Partial	No	No	No	Yes	Yes	Yes	No	Partial	69.5
	Caillon 2022 (60)	Yes	Yes	Yes	Yes	Yes	Yes	Yes	Yes	Yes	Yes	Yes	Yes	No	No	Yes	Partial	Partial	No	Yes	Yes	Yes	Yes	Yes	No	77.5
	Davison 2024 (61)	Yes	Yes	Yes	Yes	Yes	Yes	Yes	Yes	NA	Yes	Yes	Partial	Yes	Partial	Yes	Yes	No	Yes	Yes	Yes	Yes	Yes	Yes	No	83.5
	Freitag 2024 (83)	Yes	Yes	Yes	Yes	Yes	Yes	Yes	Yes	Yes	Yes	Yes	Yes	Partial	No	Yes	Yes	No	No	Yes	Yes	Yes	Yes	Yes	Partial	85.5
	Greenwood 2024 (82)	Yes	Yes	Yes	Yes	Yes	Yes	Partial	Yes	NA	NA	NA	NA	NA	NA	NA	Yes	Yes	Yes	Yes	Yes	Yes	Yes	Partial	Yes	55
	Lewkowicz 2023 (65)	Yes	Partial	Yes	Yes	Partial	Yes	Yes	Yes	Yes	Yes	Yes	Yes	Yes	Yes	Yes	Yes	No	No	Yes	Yes	Yes	Yes	Yes	No	88.5
	Liu/Zhan 2023 (90)	Yes	Yes	Yes	Yes	Yes	Yes	Yes	Yes	Yes	Yes	Yes	Partial	Partial	Yes	Partial	No	Yes	No	Yes	Yes	Yes	Yes	Yes	Yes	84
	Lin 2024 (63)	Yes	Partial	Yes	Yes	Partial	Yes	Yes	Yes	Yes	Yes	Partial	Yes	No	No	No	Partial	No	No	Yes	Yes	Yes	Yes	No	No	60
	Lin 2023 (64)	Yes	Yes	Yes	Yes	Partial	Yes	Yes	Yes	Yes	Yes	Partial	Yes	No	No	No	No	No	No	Yes	Yes	Yes	Yes	No	No	60
	Luo 2022 (71)	Yes	Yes	Yes	Yes	Yes	Yes	Yes	Yes	Yes	Yes	Yes	Yes	Yes	Yes	Yes	Yes	No	Yes	Yes	Yes	Yes	Yes	No	Yes	91
	Miranda 2022 (86)	Yes	Yes	Yes	Yes	Yes	Yes	Yes	No	Yes	NA	NA	NA	NA	NA	NA	Yes	Yes	Yes	Yes	Yes	Yes	Yes	Yes	Partial	62.5
	Morrison 2022 (76)	Yes	Yes	Yes	Yes	Partial	Yes	Yes	No	Yes	NA	Partial	Partial	No	No	Yes	Yes	Yes	No	Yes	Yes	Yes	Yes	Yes	Partial	69
	Nomura 2022 (84)	Yes	Yes	Yes	Yes	Yes	Yes	Yes	Yes	Yes	Yes	Yes	Yes	Yes	Partial	Yes	Partial	No	Yes	Partial	Yes	Yes	Yes	Yes	No	84
	Orchard 2020 (99)	Yes	Yes	Yes	Yes	No	Yes	Yes	Yes	NA	NA	NA	NA	NA	NA	NA	Yes	No	Yes	No	Yes	Yes	No	Yes	Partial	47.5
	Patel 2020 (75)	Yes	Partial	Yes	Yes	Yes	No	Yes	Yes	Partial	No	Yes	No	No	Yes	Yes	Partial	Yes	Yes	Yes	Yes	Yes	No	Partial	No	61
	Park 2024[a] (62)	Yes	Yes	Yes	Yes	No	Yes	Yes	Yes	Yes	Yes	Yes	Yes	Yes	Yes	Yes	Partial	No	Yes	Yes	Yes	Yes	Yes	Yes	No	90
	Piera-Jiménez 2020 (66)	Yes	Yes	Yes	Yes	Partial	Yes	Yes	Yes	Yes	Partial	Yes	No	No	No	Partial	No	Partial	No	No	Yes	Yes	No	No	No	55.5
	Senanayake 2023 (85)	Yes	Yes	Yes	Yes	Yes	Yes	Yes	Yes	Yes	Yes	Yes	Yes	No	Yes	Partial	Partial	Yes	Yes	Yes	Yes	Yes	Yes	Yes	Partial	86

	Velez and Malone 2021 (87)	Yes	Yes	Yes	Yes	Yes	Yes	No	Yes	Partial	Partial	Yes	Partial	Partial	No	Yes	Yes	No	No	Yes	Yes	Yes	No	No	No	62.5
	Velez 2021 (88)	Yes	Yes	Yes	Yes	Yes	Yes	No	Yes	Partial	Partial	Yes	Partial	Partial	No	Yes	Yes	No	No	Yes	Yes	Yes	No	No	No	62.5
	Wang 2021 (81)	Yes	Yes	Yes	Yes	Yes	No	No	Yes	NA	NA	NA	NA	NA	NA	NA	Yes	Yes	NA	Yes	Yes	Yes	Yes	Yes	No	49
	Zhang 2023 (59)	Yes	No	Yes	Yes	Yes	Yes	Yes	Yes	Yes	Partial	No	Partial	No	No	No	No	No	No	Yes	Yes	Yes	Yes	No	Partial	47

NA: Not applicable | EEs as a primary objective: studies in which the economic evaluation was the primary objective | EEs as a secondary objective: studies in which the economic evaluation was conducted as a secondary analysis

Assessment of reporting quality: CHEERS-(AI)

Stratum	EE first author	CHEERS-(AI) items																																						CHEERS-(AI)
		1	2	3	4	5	6	7	AI 1	8	9	10	11	12	AI 2	AI 3	AI 4	AI 5	AI 6	AI 7	13	14	15	16	17	AI 8	18	19	20	21	22	23	24	Ai9	25	26	AI 10	27	28	
EEs as a primary objective (21)	Ambrens 2022 (74)	Yes	Yes	Yes	No	Yes	Yes	Yes	NA	Yes	Yes	Yes	Yes	Yes	NA	NA	NA	NA	NA	NA	Yes	Yes	Yes	NA	NA	NA	Yes	Yes	Yes	No	Yes	Yes	Yes	NA	No	Yes	NA	Yes	Yes	89.29%†
	Bautista-Mesa 2020 (73)	Yes	Yes	Yes	No	Yes	No	Yes	NA	Yes	Yes	Yes	Yes	Yes	NA	NA	NA	NA	NA	NA	Yes	Yes	No	NA	NA	NA	No	No	Yes	No	Yes	Yes	Yes	NA	No	Yes	NA	Yes	Yes	75.00%
	Bernard 2022 (49)	No	Yes	Yes	No	Yes	No	Yes	NA	Yes	Yes	NA	Yes	Yes	NA	NA	NA	NA	NA	NA	Yes	Yes	No	NA	NA	NA	No	No	Yes	No	Yes	Yes	Yes	NA	No	No	NA	No	Yes	64.29%†
	Boggs 2022 (78)	Yes	Yes	Yes	No	Yes	Yes	No	NA	Yes	Yes	Yes	Yes	Yes	NA	NA	NA	NA	NA	NA	NA	Yes	Yes	NA	NA	NA	No	No	No	No	Yes	Yes	Yes	NA	No	Yes	NA	No	Yes	71.43%
	Buntrock 2021(57)	Yes	Yes	Yes	No	Yes	Yes	Yes	NA	Yes	Yes	NA	Yes	Yes	NA	NA	NA	NA	NA	NA	Yes	Yes	Yes	NA	NA	NA	No	No	Yes	No	Yes	Yes	Yes	NA	No	Yes	NA	No	Yes	78.57%†
	Dagenais 2021 (91)	Yes	No	Yes	No	Yes	Yes	Yes	NA	Yes	Yes	NA	Yes	Yes	NA	NA	NA	NA	NA	NA	Yes	Yes	Yes	NA	NA	NA	No	No	Yes	No	Yes	Yes	Yes	NA	No	Yes	NA	No	Yes	75.00%
	Dawkins 2024 (53)	Yes	Yes	Yes	No	Yes	No	Yes	NA	Yes	Yes	NA	Yes	Yes	NA	NA	NA	NA	NA	NA	Yes	Yes	Yes	NA	NA	NA	Yes	Yes	Yes	Yes	No	Yes	Yes	NA	Yes	Yes	NA	Yes	Yes	78.57%†
	de Jong 2020 (56)	Yes	Yes	Yes	No	Yes	Yes	No	NA	Yes	Yes	NA	Yes	Yes	NA	NA	NA	NA	NA	NA	Yes	Yes	No	NA	NA	NA	No	No	Yes	No	Yes	Yes	Yes	NA	No	Yes	NA	Yes	Yes	75.00%†
	Fatoye 2020 (54)	Yes	Yes	Yes	No	Yes	Yes	Yes	NA	Yes	Yes	NA	Yes	Yes	NA	NA	NA	NA	NA	NA	Yes	Yes	Yes	NA	NA	NA	No	No	Yes	No	Yes	Yes	No	NA	No	Yes	NA	Yes	Yes	78.57%
	van der Hout 2021 (51)	Yes	Yes	Yes	No	Yes	Yes	Yes	NA	Yes	Yes	NA	Yes	Yes	NA	NA	NA	NA	NA	NA	Yes	Yes	Yes	NA	NA	NA	No	No	Yes	Yes	Yes	Yes	Yes	NA	Yes	Yes	NA	Yes	Yes	89.29%
	Lam 2024 (93)	Yes	No	Yes	No	Yes	No	No	NA	No	Yes	No	Yes	Yes	NA	NA	NA	NA	NA	NA	Yes	Yes	Yes	NA	NA	NA	No	No	Yes	No	No	Yes	Yes	NA	No	No	NA	No	Yes	53.57%
	Lemelin 2020 (92)	Yes	No	Yes	No	Yes	No	Yes	NA	No	No	NA	Yes	Yes	NA	NA	NA	NA	NA	NA	NA	Yes	No	NA	NA	NA	No	No	No	No	No	No	No	NA	No	Yes	NA	Yes	Yes	50.00%
	Liu/Tang 2023 (89)	Yes	No	Yes	No	Yes	Yes	Yes	NA	No	Yes	NA	Yes	Yes	NA	NA	NA	NA	NA	NA	Yes	Yes	No	NA	NA	NA	No	No	Yes	No	No	Yes	Yes	NA	No	Yes	NA	No	Yes	64.29%
	Lopez-Villegas 2020 (52)	Yes	Yes	Yes	No	Yes	Yes	Yes	NA	Yes	Yes	NA	Yes	Yes	NA	NA	NA	NA	NA	NA	Yes	Yes	Yes	NA	NA	NA	No	No	Yes	No	Yes	Yes	Yes	NA	No	Yes	NA	Yes	Yes	82.14%†
	Mourad 2022 (67)	Yes	Yes	No	No	Yes	No	No	NA	No	Yes	NA	Yes	Yes	NA	NA	NA	NA	NA	NA	Yes	Yes	No	NA	NA	NA	No	No	Yes	No	Yes	Yes	Yes	NA	No	Yes	NA	No	Yes	60.71%
	Mudiyanselage 2023 (70)	Yes	Yes	No	No	Yes	No	No	NA	Yes	Yes	NA	Yes	Yes	NA	NA	NA	NA	NA	NA	Yes	Yes	Yes	NA	NA	NA	No	No	Yes	No	Yes	Yes	Yes	NA	No	Yes	NA	Yes	Yes	75.00%
	Mujcic 2022 (50)	Yes	Yes	Yes	No	Yes	No	Yes	NA	Yes	Yes	NA	Yes	Yes	NA	NA	NA	NA	NA	NA	Yes	Yes	Yes	NA	NA	NA	No	No	Yes	Yes	Yes	Yes	Yes	NA	Yes	Yes	NA	Yes	Yes	85.71%
	Nelson 2021 (68)	Yes	Yes	Yes	No	Yes	Yes	Yes	NA	Yes	Yes	NA	Yes	Yes	NA	NA	NA	NA	NA	NA	Yes	Yes	Yes	NA	NA	NA	No	No	Yes	No	Yes	Yes	Yes	NA	No	Yes	NA	Yes	Yes	82.14%
	Ney 2021 (77)	Yes	No	Yes	No	No	No	Yes	NA	No	Yes	NA	Yes	Yes	NA	NA	NA	NA	NA	NA	Yes	Yes	No	No	Yes	NA	No	No	Yes	No	Yes	Yes	Yes	NA	No	No	NA	No	Yes	53.57%
	Pelle 2022 (69)	Yes	Yes	Yes	No	Yes	No	Yes	NA	Yes	Yes	NA	Yes	Yes	NA	NA	NA	NA	NA	NA	Yes	Yes	Yes	NA	NA	NA	No	No	Yes	No	Yes	Yes	Yes	NA	No	Yes	NA	Yes	Yes	78.57%†
	Schuit 2022 (55)	Yes	Yes	Yes	No	Yes	No	Yes	NA	Yes	Yes	NA	Yes	Yes	NA	NA	NA	NA	NA	NA	Yes	Yes	Yes	NA	NA	NA	No	No	Yes	Yes	Yes	Yes	Yes	NA	Yes	Yes	NA	Yes	Yes	78.57%
EEs as a secondary objective (8)	Colomina 2021 (96)	No	Yes	No	No	Yes	Yes	Yes	NA	No	Yes	NA	Yes	Yes	NA	NA	NA	NA	NA	NA	NA	Yes	No	NA	NA	NA	No	No	No	Yes	No	Yes	No	NA	Yes	Yes	NA	Yes	Yes	64.29%
	De Batlle 2021 (95)	Yes	No	Yes	No	Yes	Yes	Yes	NA	No	Yes	NA	Yes	Yes	NA	NA	NA	NA	NA	NA	NA	Yes	No	NA	NA	NA	No	No	No	Yes	No	Yes	No	NA	Yes	Yes	NA	Yes	Yes	67.86%
	McManus 2021 (97)	No	No	Yes	No	Yes	No	Yes	NA	No	Yes	NA	Yes	Yes	NA	NA	NA	NA	NA	NA	No	Yes	No	NA	NA	NA	No	No	Yes	Yes	Yes	Yes	Yes	NA	Yes	Yes	NA	No	Yes	64.29%
	Park 2023 (94)	No	No	No	No	Yes	No	Yes	No	No	Yes	NA	Yes	Yes	Yes	NA	Yes	Yes	Yes	No	No	No	No	No	NA	NA	No	No	No	No	No	Yes	No	No	No	Yes	Yes	No	Yes	44.74% (AI)

	Priebe 2024 (79)	Yes	No	Yes	No	Yes	Yes	Yes	NA	Yes	Yes	NA	Yes	Yes	NA	NA	NA	NA	NA	NA	NA	No	No	NA	NA	NA	No	No	Yes	No	Yes	Yes	Yes	NA	No	Yes	NA	Yes	Yes	71.43%
	Smak-Gregoor 2023 (98)	No	Yes	No	No	Yes	Yes	No	Yes	No	Yes	NA	Yes	Yes	Yes	NA	No	Yes	Yes	No	NA	No	Yes	NA	NA	No	No	No	Yes	No	No	Yes	Yes	Yes	No	Yes	No	No	Yes	57.89% (AI)
	Sten-Gahmberg 2024 (58)	No	No	Yes	No	Yes	Yes	Yes	NA	Yes	Yes	NA	Yes	Yes	NA	NA	NA	NA	NA	NA	Yes	Yes	Yes	NA	NA	NA	No	Yes	Yes	Yes	No	Yes	Yes	NA	Yes	Yes	NA	Yes	Yes	82.14%†
	Taylor 2020 (80)	NA	Yes	Yes	No	Yes	Yes	Yes	NA	No	Yes	NA	Yes	Yes	NA	NA	NA	NA	NA	NA	Yes	Yes	Yes	NA	NA	NA	Yes	No	Yes	Yes	Yes	Yes	Yes	NA	Yes	Yes	NA	Yes	Yes	89.29%†
**Model-based EEs (22)**	Bhardwaj 2021 (72)	Yes	Yes	Yes	No	Yes	Yes	Yes	NA	Yes	Yes	NA	Yes	Yes	NA	NA	NA	NA	NA	NA	Yes	Yes	Yes	Yes	Yes	NA	No	No	Yes	No	Yes	Yes	Yes	NA	No	Yes	NA	No	Yes	78.58%
	Caillon 2022[c,] (60)	Yes	No	Yes	No	Yes	No	No	NA	Yes	Yes	Yes	Yes	Yes	NA	NA	NA	NA	NA	NA	Yes	Yes	Yes	Yes	Yes	NA	Yes	Yes	Yes	No	Yes	Yes	Yes	NA	No	Yes	NA	Yes	Yes	78.57%†
	Davison 2024 (61)	Yes	No	Yes	No	Yes	Yes	No	NA	Yes	Yes	Yes	Yes	Yes	NA	NA	NA	NA	NA	NA	Yes	Yes	Yes	Yes	Yes	NA	Yes	No	Yes	No	Yes	Yes	Yes	NA	No	Yes	NA	Yes	Yes	78.57%†
	Freitag 2024[b] (83)	Yes	No	Yes	No	Yes	Yes	No	NA	Yes	Yes	Yes	Yes	Yes	NA	NA	NA	NA	NA	NA	NA	Yes	No	Yes	Yes	NA	NA	NA	Yes	No	Yes	Yes	Yes	NA	No	Yes	NA	Yes	Yes	78.57%†
	Greenwood 2024 (82)	Yes	No	No	Yes	Yes	Yes	Yes	NA	Yes	Yes	NA	Yes	Yes	NA	NA	NA	NA	NA	NA	Yes	Yes	Yes	No	Yes	NA	No	No	Yes	Yes	Yes	Yes	Yes	NA	Yes	Yes	NA	Yes	Yes	82.14%†
	Lewkowicz 2023 (65)	Yes	Yes	Yes	No	No	Yes	Yes	NA	Yes	Yes	Yes	Yes	Yes	NA	NA	NA	NA	NA	NA	Yes	Yes	Yes	Yes	Yes	NA	No	No	Yes	No	Yes	Yes	Yes	NA	No	Yes	NA	No	Yes	75.00%
	Liu/Zhan 2023 (90)	Yes	Yes	Yes	No	Yes	No	Yes	NA	No	Yes	Yes	Yes	Yes	NA	NA	NA	NA	NA	NA	Yes	Yes	Yes	Yes	Yes	NA	No	No	Yes	No	Yes	Yes	Yes	NA	No	Yes	NA	No	Yes	71.43%
	Lin 2024[d] (63)	Yes	No	Yes	No	Yes	Yes	Yes	Yes	Yes	Yes	Yes	Yes	Yes	Yes	NA	No	Yes	Yes	No	NA	Yes	Yes	Yes	Yes	NA	No	No	Yes	No	Yes	Yes	Yes	No	No	Yes	Yes	No	Yes	73.68% (AI)
	Lin 2023[d] (64)	Yes	Yes	Yes	No	Yes	Yes	Yes	Yes	Yes	Yes	Yes	Yes	Yes	Yes	NA	No	Yes	Yes	No	Yes	Yes	Yes	Yes	Yes	NA	No	No	Yes	No	Yes	Yes	Yes	No	No	Yes	Yes	No	Yes	76.32% (AI)
	Luo 2022 (71)	Yes	Yes	No	No	Yes	No	Yes	NA	Yes	Yes	Yes	Yes	NA	NA	NA	NA	NA	NA	NA	NA	Yes	Yes	Yes	Yes	NA	No	No	Yes	No	Yes	Yes	Yes	NA	No	Yes	NA	No	Yes	71.43%†
	Miranda 2022 (86)	Yes	Yes	Yes	No	Yes	Yes	Yes	NA	Yes	Yes	No	Yes	Yes	NA	NA	NA	NA	NA	NA	Yes	Yes	Yes	No	Yes	NA	No	No	Yes	No	Yes	Yes	Yes	NA	No	Yes	NA	No	Yes	71.43%
	Morrison 2022 (76)	Yes	Yes	Yes	No	Yes	Yes	No	No	Yes	Yes	Yes	Yes	Yes	Yes	No	No	No	Yes	No	Yes	Yes	Yes	Yes	Yes	NA	No	No	Yes	NA	Yes	Yes	Yes	Yes	No	Yes	No	Yes	Yes	71.05% (AI) †
	Nomura 2022 (84)	Yes	Yes	No	No	Yes	No	Yes	NA	Yes	Yes	Yes	Yes	Yes	NA	NA	NA	NA	NA	NA	Yes	Yes	Yes	Yes	Yes	NA	No	No	Yes	No	Yes	Yes	Yes	NA	No	Yes	NA	Yes	Yes	75.00%†
	Orchard 2020 (99)	Yes	No	No	No	Yes	Yes	Yes	NA	No	Yes	No	Yes	No	NA	NA	NA	NA	NA	NA	No	Yes	Yes	No	No	NA	No	No	No	No	Yes	Yes	No	NA	No	Yes	NA	No	Yes	42.86%
	Patel 2020 (75)	Yes	Yes	No	Yes	Yes	No	No	NA	Yes	Yes	Yes	Yes	Yes	NA	NA	NA	NA	NA	NA	NA	Yes	Yes	Yes	Yes	NA	No	No	Yes	No	Yes	Yes	Yes	NA	No	No	NA	No	Yes	67.86%†
	Park 2024[a] (62)	Yes	Yes	Yes	No	Yes	No	Yes	NA	Yes	Yes	Yes	Yes	Yes	NA	NA	NA	NA	NA	NA	Yes	Yes	Yes	Yes	Yes	NA	No	No	Yes	No	Yes	Yes	Yes	NA	No	Yes	NA	Yes	Yes	78.57%†
	Piera-Jiménez 2020 (66)	Yes	No	Yes	No	Yes	Yes	Yes	NA	Yes	Yes	Yes	Yes	Yes	NA	NA	NA	NA	NA	NA	No	Yes	Yes	Yes	Yes	NA	No	No	Yes	No	Yes	Yes	Yes	NA	No	Yes	NA	Yes	Yes	75.00%†
	Senanayake 2023 (85)	Yes	No	Yes	No	Yes	Yes	Yes	NA	Yes	Yes	Yes	Yes	Yes	NA	NA	NA	NA	NA	NA	NA	Yes	Yes	Yes	Yes	NA	No	No	Yes	No	Yes	Yes	Yes	NA	No	Yes	NA	No	Yes	75.00%†
	Velez and Malone 2021 (87)	Yes	Yes	Yes	No	No	No	Yes	NA	Yes	Yes	NA	Yes	Yes	NA	NA	NA	NA	NA	NA	No	Yes	Yes	Yes	No	NA	No	No	Yes	No	Yes	Yes	Yes	NA	No	Yes	NA	Yes	Yes	67.86%
	Velez 2021 (88)	Yes	No	Yes	No	No	No	Yes	NA	Yes	Yes	NA	Yes	Yes	NA	NA	NA	NA	NA	NA	NA	Yes	No	Yes	Yes	NA	No	No	Yes	No	Yes	Yes	Yes	NA	No	Yes	NA	No	Yes	64.29%
	Wang 2021 (81)	Yes	No	Yes	No	No	No	Yes	NA	Yes	Yes	NA	Yes	Yes	NA	NA	NA	NA	NA	NA	NA	Yes	Yes	No	Yes	NA	No	No	Yes	No	No	Yes	Yes	NA	No	Yes	NA	Yes	Yes	64.29%
	Zhang 2023[e] (59)	No	No	No	No	Yes	No	Yes	NA	No	Yes	Yes	No	No	NA	NA	NA	NA	NA	NA	No	Yes	NA	Yes	Yes	NA	Yes	No	No	Yes	No	Yes	Yes	NA	NA	No	NA	No	Yes	53.63% (AI)

NA: Not applicable | †: Use of reporting guidelines | EEs as a primary objective: studies in which the economic evaluation was the primary objective | EEs as a secondary objective: studies in which the economic evaluation was conducted as a secondary analysis

Appendix 6: Prompt used to support CHEERS-(AI) assessment

NOTE: WAS NOT USED TO FINALLY ASSESS THE QUALITY BUT FOR LOW-THRESHOLD SUPPORT

Task: You are an expert in health economic evaluation methodology. Your job is to assess the reporting quality of a study based on the CHEERS 2022 checklist. Instructions: Extract Information: Identify whether the study explicitly reports on each of the 28 CHEERS items. Assess Completeness: Determine if the information provided is fully reported, partially reported, or missing for each checklist item.

Step 1: Please answer each of the following questions and provide a reasoning for your answer. Also provide the page and/or section in which you found the answer.

1. Title – Does the paper's title clearly state that it is an economic evaluation and detailing which interventions or strategies are being compared, so that the study is easily identifiable and indexable? Report a "yes" even if the comparator is not stated.
2. Abstract – Does the abstract provide a structured and comprehensive summary that includes the study's context, objectives, key methods (including the study population, setting, perspective, and time horizon), main results (with costs and outcomes), and any alternative analyses or sensitivity checks, in a manner that allows rapid screening and initial quality appraisal?

3. Background and Objectives – Does the introduction clearly describe the decision problem or policy context motivating the study, specify the study question, and explain the relevance and potential impact on patients, stakeholders, or health policy, including any prespecified subgroups or decision-relevant aspects? Its important that the research question and/or the decision problem is in line with items 5 and 8 (setting/location and perspective).

4. Health Economic Analysis Plan – Is there a statement indicating that a health economic analysis plan (HEAP) was developed, with details provided about its availability (for example, as supplementary material or in a repository) to ensure transparency and mitigate reporting bias?

5. Study Population – Does the paper describe in detail the characteristics of the study population (such as age range, gender, socioeconomic status, clinical characteristics, etc.) and, if applicable, any subgroups or risk factors that may influence costs and outcomes, thereby facilitating generalizability and targeted interpretation?

6. Setting and Location – Does the study provide detailed contextual information about the geographical, institutional, or system-level setting (e.g., country, healthcare sector, payment scheme) in which the evaluation was conducted, allowing the reader to assess external validity and transferability of results? It is not sufficient just to state the location. There needs to be a bit more information. This could be that the location is in a rural area or information about how many potential participants live in that area (that are just examples to describe what kind of information about the setting are required, meaning there could be other contextual information about the setting).

7. Comparators – Are the interventions or strategies compared described in sufficient detail (including dosage, frequency, intensity, or complexity of the intervention) along with a clear rationale for why these comparators were chosen, including any justification for excluding other alternatives?

8. Perspective – Does the paper clearly state the perspective(s) (e.g., healthcare payer, societal, patient) adopted in the analysis, provide a description of which costs and outcomes are included under that perspective, and justify why that perspective is appropriate given the decision context?

9. Time Horizon – Is the time horizon over which costs and outcomes are measured clearly reported and justified (e.g., capturing all relevant long-term effects or costs), and does the study discuss its impact on the findings, particularly in cases where benefits or harms occur over extended periods?

10. Discount Rate – Does the paper report the discount rate(s) applied to future costs and outcomes, including a justification (such as referencing local guidelines or the nature of the intervention), and discuss how variations in the discount rate might affect the study's results? Discount rates are only applicable for studies with a time horizon > 1 year.

11. Selection of Outcomes – Are the outcomes chosen for measuring benefits and harms clearly described, including their definitions and relevance to the decision problem, and is the rationale for selecting these outcomes (including any composite measures) provided?

12. Measurement of Outcomes – Does the study detail how outcomes (benefits and harms) were measured, including the instruments or methods used (e.g., clinical scales, quality-adjusted life years [QALYs]), and clarify whether these methods are validated and appropriate for the population studied?

13. Valuation of Outcomes – Is there a clear description of the methods used to assign value to outcomes (such as utility measurement methods, willingness-to-pay approaches, or mapping techniques), including details on the study population from which these values were derived and any assumptions or adjustments made? This is only applicable for preference-based outcomes.

14. Measurement and Valuation of Resources and Costs – Does the paper explain how resources and costs were identified, measured, and valued (including whether microcosting or gross-costing methods were used), and is there transparency about the data sources and any adjustments (e.g., for opportunity costs) applied in the valuation process?

15. Currency, Price Date, and Conversion – Are the dates corresponding to cost estimates, the currency used (with appropriate ISO codes), and any conversion or inflation adjustments clearly reported, so that readers can accurately interpret cost data in context?

16. Rationale and Description of Model (if applicable) – If modeling is used, does the paper provide a detailed description of the model structure (including diagrams or flowcharts if available), explain why modeling was necessary, discuss the assumptions and data sources underpinning the model, and indicate whether the model is publicly accessible?

17. Analytics and Assumptions (only applicable for model based evaluations) – Are the analytical methods for data transformation, extrapolation, and validation of models or statistical techniques clearly described, including an explanation of the underlying assumptions (with references or justifications) and any sensitivity or uncertainty analyses performed?

18. Characterizing Heterogeneity – Does the study describe methods to analyze how results vary across different subgroups (e.g., by age, gender, risk factors), including both relative and absolute differences, and provide justification for the chosen approach (or, if assuming homogeneity, explain why this is reasonable)?

19. Characterizing Distributional Effects – Is there a description of how the study addresses equity or distributional issues (for example, by adjusting for socioeconomic status, geographical variations, or special populations), and are any methods used to capture differential impacts or to modify thresholds for equity considerations clearly outlined?

20. Characterizing Uncertainty – Does the paper describe in detail the methods used to explore uncertainty in the analysis (such as probabilistic sensitivity analyses, deterministic sensitivity analyses, bootstrapping, or use of cost-effectiveness acceptability curves), and does it explain how uncertainty in parameters or structural assumptions might influence the conclusions?

21. Approach to Engagement with Patients and Stakeholders – Are the methods for engaging patients, service recipients, the public, or other stakeholders (such as clinicians or payers) in the design or conduct of the study clearly reported, including details on the nature and impact of such involvement on study design, outcome selection, or interpretation?

22. Study Parameters – Does the paper provide a comprehensive list or table of all analytic inputs (including values, ranges, sources, and assumptions used in the model or analysis), so that another researcher could replicate the analysis, and is the uncertainty or variability of these parameters reported?

23. Summary of Main Results – Are the key results summarized clearly, including the mean values (both discounted and undiscounted, where appropriate) for the main cost and outcome categories, and is the overall summary measure (e.g., incremental cost-effectiveness ratio) reported in a way that facilitates comparison across studies?

24. Effect of Uncertainty – Does the study explicitly discuss how uncertainty in analytic judgments, inputs, or model projections affects the main findings (including an assessment of how variations in discount rate or time horizon impact results), and are these effects illustrated using appropriate graphs or tables?

25. Effect of Engagement – If applicable, does the paper report on how the involvement of patients, the public, or other stakeholders influenced the study's approach, methods, or findings, providing specific examples of changes made as a result of this engagement?

26. Discussion of Findings, Limitations, and Generalizability – In the discussion section, does the paper critically appraise its own findings by summarizing key results, acknowledging limitations (including ethical or equity issues not captured in the analysis), and discussing how these might affect the application of the results in different policy or practice settings?

27. Source of Funding – Is there a clear statement describing how the study was funded, including the role (if any) that the funder played in the study's design, conduct, analysis, or reporting, to assess potential conflicts or biases?

28. Conflicts of Interest – Does the paper report all potential conflicts of interest for the authors, in accordance with the journal or International Committee of Medical Journal Editors requirements, ensuring transparency in the study's conduct and reporting?

Step 2: Be critical but fair with your assessment. Please check every yes twice and make sure, that the main points of each question are fullfilled.

Step 3: Provide a table with yes if the asked information are fulfilled, a N/A if the question is not applicable (e.g. if the question is about models used in the paper but the paper is not model-based) and a no if the asked information is not fulfilled or not fully fulfilled.

Please have in mind that we write a rapid review which will be published. Thus, we need to be sure that the information are correct. Please self-verify your answer.
